# Cerebral amyloid angiopathy-related cardiac injury: Focus on cardiac cell death

**DOI:** 10.3389/fcell.2023.1156970

**Published:** 2023-02-24

**Authors:** Xiaofang Xu, Huikang Xu, Zhaocai Zhang

**Affiliations:** ^1^ Department of Critical Care Medicine, The Second Affiliated Hospital, Zhejiang University School of Medicine, Hangzhou, Zhejiang, China; ^2^ Key Laboratory of the Diagnosis and Treatment for Severe Trauma and Burn of Zhejiang Province, Hangzhou, China; ^3^ Zhejiang Province Clinical Research Center for Emergency and Critical care medicine, Hangzhou, China

**Keywords:** cerebral amyloid angiopathy, cardiac injury, cardiac amyloidosis, amyloid β, alzheimer’s disease

## Abstract

Cerebral amyloid angiopathy (CAA) is a kind of disease in which amyloid β (Aβ) and other amyloid protein deposits in the cerebral cortex and the small blood vessels of the brain, causing cerebrovascular and brain parenchymal damage. CAA patients are often accompanied by cardiac injury, involving Aβ, tau and transthyroxine amyloid (ATTR). Aβ is the main injury factor of CAA, which can accelerate the formation of coronary artery atherosclerosis, aortic valve osteogenesis calcification and cardiomyocytes basophilic degeneration. In the early stage of CAA (pre-stroke), the accompanying locus coeruleus (LC) amyloidosis, vasculitis and circulating Aβ will induce first hit to the heart. When the CAA progresses to an advanced stage and causes a cerebral hemorrhage, the hemorrhage leads to autonomic nervous function disturbance, catecholamine surges, and systemic inflammation reaction, which can deal the second hit to the heart. Based on the brain-heart axis, CAA and its associated cardiac injury can create a vicious cycle that accelerates the progression of each other.

## 1 Introduction

Cerebral amyloid angiopathy (CAA) is a kind of neurological disease in which amyloid substances deposition in the cerebral cortex and the wall of small and medium blood vessels leads to vascular dysfunction and brain parenchymal injury. CAA is prevalent in the elderly, especially in old people with Alzheimer’s disease (AD). In the community, about a quarter of the elderly over 70 years old suffer from CAA, and people over 85 have a 70%–80% chance of having CAA. In patients with AD, the incidence of CAA is up to 90% (Arvanitakis et al., 2011). As a familiar age-related vascular disease, CAA can bring about multiple organ dysfunction and focal neurological impairment with rapid progression. Neurological impairment usually induces epilepsy and cognitive impairment further (Viswanathan and Greenberg, 2011). CAA is a common etiology of cerebral hemorrhage, and CAA-related intracranial hemorrhage (CAA-RICH) can aggravate the disturbance of motor function, higher cortical function and consciousness of the brain. In the SMASH-U etiological classification system of intracerebral hemorrhage, CAA-RICH accounts for about 20% of all intracerebral hemorrhage, second only to hypertensive intracerebral hemorrhage (Mosconi et al., 2021; Jia et al., 2022). In addition to causing cerebral hemorrhage, the pathogenetic process of CAA is often accompanied by cardiac injury (Gagno et al., 2020).

The brain is the higher center of the nervous system, which regulates the functions of various organs and receives feedback from the whole body. Both clinical and experimental evidence suggests that there is a causal correlation between brain injury and cardiac injury due to the exist of brain-heart axis. CAA-related brain injury involving the heart may result in severe cardiac injury (such as heart failure, myocardial infarction, neurogenic sudden cardiac death, etc.), or slightly reversible heart injury (such as myocardial injury, transient arrhythmia, neurogenic stress cardiomyopathy, etc.) (Chen et al., 2017; Sposato et al., 2020). A damaged heart, in turn, affects blood flow to the brain, exacerbating existing or new brain damage. Aβ, a key protein in CAA, is involved in plaque rupture, thrombosis, and acute coronary syndrome (Stakos et al., 2020). Moreover, Aβ1-40 accumulation in the blood, blood vessel walls, and cardiac tissue may contribute to myocardial injury, cardiovascular lesions, and heart failure (Roeben et al., 2016; Bayes-Genis et al., 2017; Stamatelopoulos et al., 2018). AD often co-occurs with heart failure and the two diseases shares common risk factors, hypercholesterolemia and metabolic syndrome (Chhatre et al., 2009; Rusanen et al., 2014; Cermakova et al., 2015). Cerebral amyloid angiopathy-related cardiac injury (CAA-CI) is a new research field, which needs to analyze in depth. The article reviews the common pathogenesis of CAA and cardiac amyloidosis (CA) and the influence of CAA on heart injury.

## 2 Comorbid mechanisms of CAA and CA

AD/CAA and CA have long been considered distinct diseases, but they share the same risk factors and similar epidemiological stratification (Stakos et al., 2020). In a retrospective analysis, clinical and transthoracic echocardiographic data from AD and non-AD subjects of the same age and sex showed that patients with AD may have left ventricular hypertrophy and heart failure with preserved ejection fraction (HFpEF) (Johansen et al., 2020). The results indicate that there may be an underlying connection between AD and cardiac disease. According to current evidence as follows, Aβ, microtubule-associated protein tau and transthyretin amyloidosis (ATTR) may be the key proteins in bridging CAA and CA.

### 2.1 The role of Aβ in the pathogenesis of CAA/AD and CA

Generally, Aβ is mainly divided into Aβ1-40 and Aβ1-42 peptide, which is produced by abnormal processing of amyloid precursor protein (APP) (Cisternas et al., 2019). Extracellular aggregates of Aβ plagues are a key factor in the development of AD (Tiwari et al., 2019). These amyloid proteins in cerebrovascular originate from neurons. They drain from the perivascular interstitium of the brain parenchyma and leptomeninges, and are finally deposited along the blood vessels (Weller et al., 2008). Aβ deposits in the brain trigger a series of neurological dysfunction events, such as cognitive decline and progressive AD dementia. Amyloid plaques in the brain vary according to the Aβ combination form. Neuroinflammatory plaques were caused by Aβ1-40 and Aβ1-42, while diffuse plaques were mainly composed of Aβ1-42. In AD patients, a large amount of Aβ deposition was not only found in the brain tissue, but also in cardiomyocytes and myocardial interstitium (Troncone et al., 2016). Moreover, the presence of Aβ1-40 and Aβ1-42 has been shown to be associated with myocardial diastolic dysfunction (Troncone et al., 2016). In addition, Aβ preamyloid oligomers (PAOs) have also been shown to have toxic effects on cardiomyocytes (Gianni et al., 2010). Cardiac abnormalities in AD patients are characterized by increased ventricular septal thickness, diastolic dysfunction, and electrocardiograph (ECG) changes, such as decreased QRS voltage or voltage/mass ratio (Sanna et al., 2019).

Aβ aggregates not only appear in brain and heart, but also accumulate in the blood vessel wall and cause damage to other organs and tissues (Kuo et al., 2000). Aβ deposition in blood vessels induces and promotes vascular inflammation and atherosclerosis (Howlett et al., 2019; Kang et al., 2021; Kapasi et al., 2021). Vessels for Aβ deposition include leptomeningeal and cortical vessels in CAA, cerebral microvessels, intracerebral arteries/Willis, aorta, and coronary/extracerebral arteries (Stakos et al., 2020). Aβ deposits can be found in the cardiac tissue of AD patients, and these deposits are related to the pathogenesis of CA (Schaich et al., 2019). From the perspective of tissue distribution, the accumulation of Aβ in the brain tissues of patients with AD or CAA is much higher than that in the heart of CA patients (Troncone et al., 2016). Aβ-induced cardiovascular disease, in turn, leads to cerebral hypoperfusion, which is a risk factor for vascular dementia, AD, or combined dementia (Debette et al., 2007). The researchers also proposed that AD is actually a multi-organ disease with Aβ circulating in the blood, which can affect both the heart and the brain (Troncone et al., 2016).

### 2.2 The role of tau protein between CAA and CA

In addition to amyloid plaques composed of Aβ1-40 and Aβ1-42 peptides, neurofibrillary tangle (NFT)s composed of the hyperphosphorylated microtubule-associated protein tau are another important pathological feature of AD (Sun et al., 2021). Abnormal tau aggregation can be detected in patients with endothelial dysfunction and cognitive impairment in the brain, which is often accompanied by damage to the blood-brain barrier (BBB) (Kim et al., 2018). The permeability of the BBB increasing will further aggravate the accumulation of tau, resulting in a vicious cycle of vascular and cerebral parenchymal injuries (Blair et al., 2015; Merlini et al., 2016). Both Aβ and tau are hallmark proteins of AD pathogenesis, and they are closely related. Aβ can induce tau hyperphosphorylation, and hyperphosphorylated tau mediates the toxicity of Aβ, both of which can amplify the toxicity of each other (Godemann et al., 1999; Hernandez et al., 2009; Iijima et al., 2010). In many studies, amyloid protein and tau pathology are interdependent and coexist, and tau pathology is inextricably linked with CAA and CA lesions (Ghetti et al., 1996; Vidal et al., 2000a; Oshima et al., 2008). Moreover, studies have shown that endogenous wild-type tau protein can cause Aβ accumulation and cognitive defects in AD mouse models (Roberson et al., 2007; Andrews-Zwilling et al., 2010; Roberson et al., 2011). Tau also exists in amyloid deposits of sporadic inclusion body myositis, co-causing disease with amyloid protein (Askanas et al., 1994). Nevertheless, its effect on amyloid formation remains unclear.

Studies have shown that the genetic variation of tau may lead to senile systemic amyloidosis and further cause myocardial infarction, indicating that tau is associated with CA (Tanskanen et al., 2008). Researchers found the presence of Aβ and tau proteins in ultrastructure tests of heart tissue biopsies from patients with CA (Fidziańska et al., 2011). Immunohistochemical tests of the amyloidosis hearts showed that Aβ and tau can form amyloid aggregates, which further lead to myocardial damage and cardiac dysfunction (Fidziańska et al., 2011). In addition, ischemia-reperfusion injury of the heart can also promote tau hyperphosphorylation and mitochondrial dysfunction in the brain, aggravating cerebral damage (Leech et al., 2020). Amyloidosis is an age-related systemic disease, in which tau and Aβ pathology can affect myocardium and brain neurovascular at the same time. Aβ deposits and the formation of NFTs often lead to the cardiac comorbidities of AD patients, including amyloid cardiomyopathy and cardiovascular disease, eventually leading to multiple organ failure (Shityakov et al., 2021).

### 2.3 The interaction between CA and CAA

Transthyroxine (TTR) is the main carrier of thyroxine (T4) in cerebrospinal fluid and blood. Wild-type transthyroxine (ATTRwt) is an amyloid precursor found in heart deposits in the old. Aggregation of cardiac TTR amyloid protein can lead to myocardial fibrosis and heart failure (Hamasaki et al., 2022). TTR amyloid protein in cerebrospinal fluid (CSF) has been shown to bind directly to Aβ, blocking the progression of AD (Buxbaum et al., 2008; Silva et al., 2017). According to a Japanese population-based study of 240 successive autopsy cases, cardiac ATTR deposition was relevant to AD cerebral pathologies in patients≤90 years at death, indicating a close relationship between CA and AD (Hamasaki et al., 2022). At present, no significant correlation has been found between cardiac TTR amyloid deposition and CAA staging. Intriguingly, the pathogenesis of CA is mainly due to the deposition of ATTR, which is closely related to CAA, but ATTR can prevent the progression of CAA. We speculate that pathogenesis of CAA is due not only to the Aβ deposits, but also to ATTR and other substances deposits, which contributing to the contradiction. Obviously, the variation and deposition of ATTR itself is another important etiology of CAA.

ATTR is an amyloid protein synthesized mainly by the liver, choroid plexus and retinal pigment epithelium. ATTR, an important mediator of CA, has been recognized as the cause of HFpEF in the elderly, accounting for 13% of HFpEF (González-López et al., 2015). The ATTR variants widely exist in patients with familial amyloid polyneuropathy and familial amyloid cardiomyopathy, which can also be detected by Pittsburgh compound B (PIB)- positron emission tomography (PET) in the leptomeningeal vessels of cerebrum, cerebellum, brainstem, and spinal cord of CAA patients (Cavallaro and Klunk, 2016). CAA is likely to be a late manifestation of familial amyloid polyneuropathy with ATTR, but whether CA can directly affect the pathology of CAA is not clear currently. Liver transplantation has a significant short-term effect on ATTR-associated CAA, indicating that this type of CAA is mainly caused by ATTR produced by the liver (Yamashita et al., 2008). Liver-derived variants of ATTR can cause CA and CAA by reaching the heart and leptomeninges *via* blood circulation and cerebrospinal fluid (Park et al., 2019). In conclusion, when amyloid cardiomyopathy occurs, ATTR from the heart or bloodstream may enter the cerebrospinal fluid through ruptured BBB and accelerate the progression of CAA.

### 2.4 Co-pathogenesis of CAA/AD and CA based on Aβ/tau/ATTR

**FIGURE 1:**
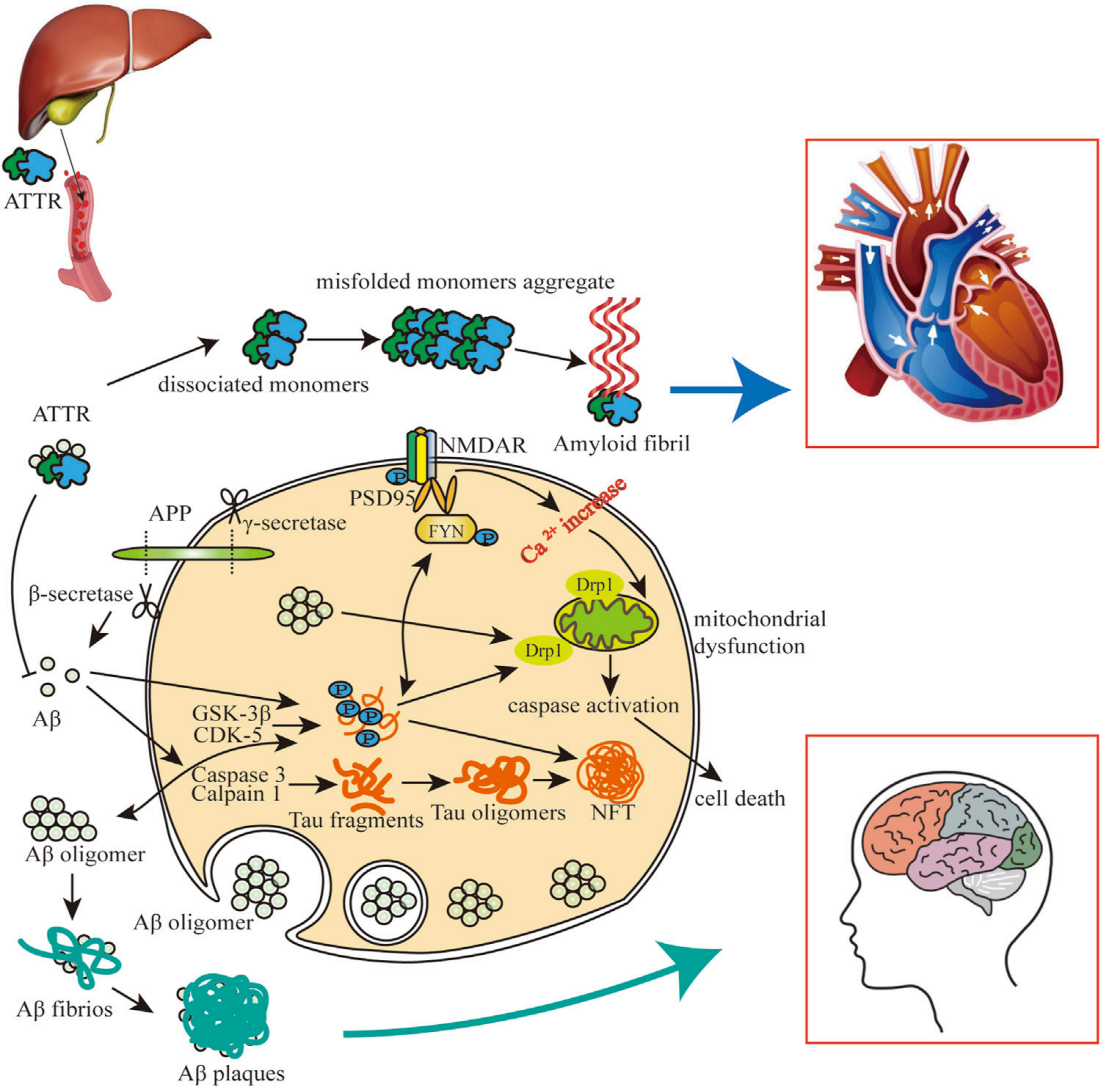
The impact of Aβ-tau-ATTR on AD/CAA and CA. Aβ and tau can enter cells and their intracellular interaction can cause mitochondrial dysfunction and brain/heart cell death. Highly phosphorylated tau promotes extracellular aggregation of Aβ, and extracellular aggregation of Aβ and ATTR contributes to amyloidosis in heart and brain. Aβ plaques and NFTs mainly participated in the pathology of CAA and AD, and ATTR amyloid fibrils are mainly involved in the pathology of CA.

In the progression of AD, extracellular amyloid plaques and NFTs are characteristic pathogenic hallmarks. Aβ can promote the production of tau, which is also an important medium for Aβ to play a toxic role. Aβ drives tau hyperphosphorylation by activating cyclin-dependent kinase (CDK)-5 and glycogen synthase kinase (GSK)-3β, and Aβ promotes the cleavage of caspase3 into tau fragments (Gamblin et al., 2003; Terwel et al., 2008; Hernandez et al., 2009). Aβ and tau act together to produce adverse impacts on mitochondria, which lead to reactive oxygen species (ROS) aggregating, adenosine triphosphate (ATP) synthesis and Ca^2+^ regulation abnormalities, and ultimately cell death (Kang et al., 2011). ATTR is mainly produced by the liver, and it can bind directly to Aβ to decrease the aggregation of Aβ and mitigate the progression of AD/CAA (Silva et al., 2017). ATTR itself can also form amyloid fibrils due to misfolding, causing heart and brain damage (Lamb, 2021) (Figure 1).

## 3 The “two-hits” mechanism of CAA-related cardiac injury

Given that CAA shares same pathophysiological basis with AD and CA, CAA may promote the formation of cardiac vulnerability. We believe that it is necessary to attach importance to CAA-related cardiac injury (CAA-CI) as a special type of cardiac disease from the perspective of pathophysiology. The effects of CAA on the heart can be divided into two parts. Early in the onset of CAA, its related pathological processes can affect the heart. When CAA progresses to cerebral hemorrhage, stroke can cause secondary damage to cardiac tissue.

### 3.1 Mechanism of “first hit” of CAA on heart (pre-stroke)

**FIGURE 2:**
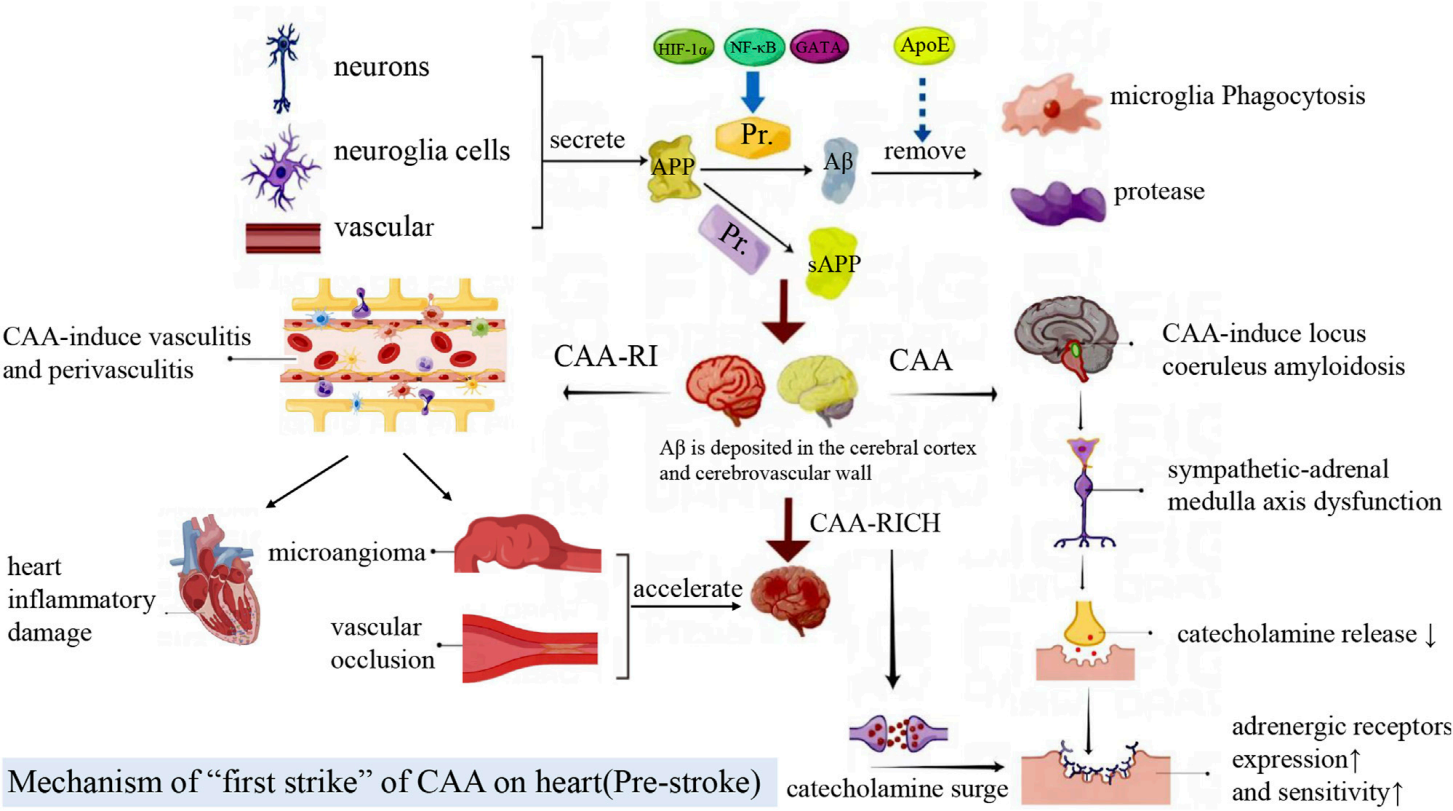
Amyloid precursor protein (APP) is mainly produced by neurons, glial cells and blood vessels. It is cleaved by enzymes to produce Aβ or sAPP fragments, which are cleared by macrophages and proteases. Aβ is deposited in the brain parenchyma, blood vessels and cells. Locus coeruleus amyloidosis impairs the sympathetic-adrenal medulla axis and cardiac autonomic function, then further leads to the increased adrenergic receptor expression and activity, which exacerbates heart injury after CAA-RICH. CAA-related vasculitis and perivasculitis can lead to inflammatory damage in the heart. At the same time, CAA-RI increases vascular fragility and conversely accelerates the CAA-RICH process.

CAA can cause initial damage to the heart before the occurrence of CAA-RICH. When CAA causes sympathetic-adrenal medulla axis disorder and local inflammation, it generates adverse effects on the heart. In addition, circulating Aβ of CAA patients can also aggravate CAA-CI. These factors all bring about CAA-related “first hit” (Figure 2).

#### 3.1.1 CAA causes sympathetic–adrenal medulla axis dysfunction

CAA is often accompanied by AD pathogenesis, and 90% AD patients suffer from CAA (Arvanitakis et al., 2011). Locus coeruleus (LC) is one of the first brain regions to undergo degeneration in AD, and it is also the central site of the sympathetic adrenal medulla system (Ross et al., 2019). LC amyloidosis can cause damage to the sympathetic - adrenal medulla axis and dysfunction of cardiac autonomic nerve (Szabadi, 2013). The LC degeneration and sympathetic axis injury in CAA can lead to decreased secretion of catecholamine, which induce the adrenergic receptors increased expression and sensitivity in peripheral tissues and cells. The catecholamine surge will bring about greater heart damage when other stressful situations occurred (Gannon et al., 2015).

#### 3.1.2 CAA mediates cardiac inflammatory injury

In early CAA patients, CAA is often complicated by the vasculitis and perivasculitis, that is, inflammatory reaction of macrophages, microglia and lymphocytes infiltration in the endovascular walls or perivascular spaces (Salvarani et al., 2013). Cerebral pro-inflammatory cytokines and toxic Aβ reach the heart through damaged endothelial cells and the BBB, further inducing systemic inflammatory response and cardiac inflammatory damage (Shishehbor et al., 2007; Tublin et al., 2019). CAA-related inflammation also causes pathological changes such as endovascular lumen formation, vascular occlusion, vascular hyalinosis, microangioma dilatation and fibrinoid necrosis (Mandybur, 1986). These changes result in increased vascular brittleness, cerebral infarction, cerebral ischemia or fatal cerebral lobular hemorrhage, then accelerate the CAA-RICH process (Arvanitakis et al., 2011).

#### 3.1.3 Cardiac damage mediated by Aβ and its precursors

**FIGURE 3:**
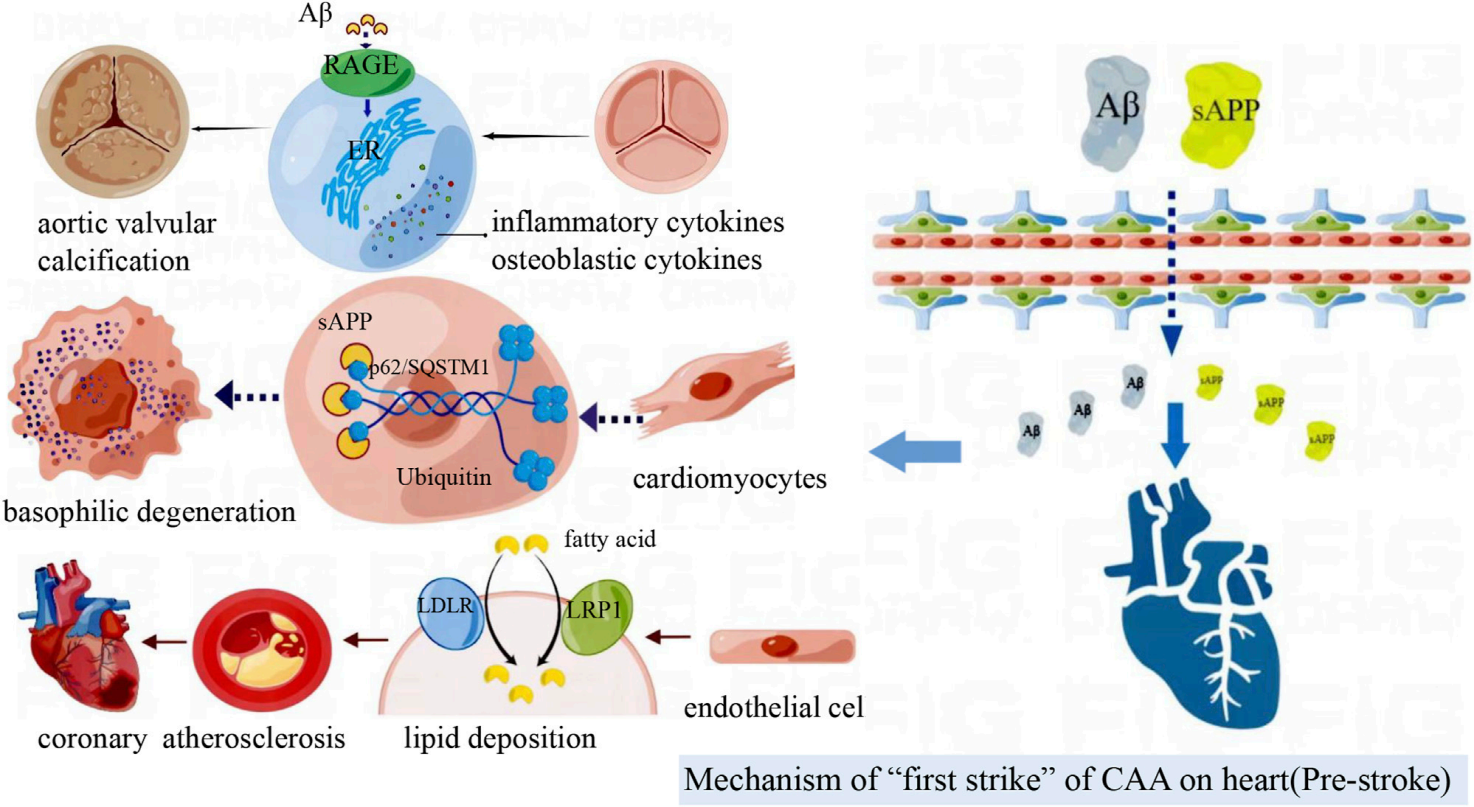
Amyloid deposited in the brain can cross the blood-brain barrier to reach the peripheral vasculature and heart. Aβ deposition in blood vessels accelerate coronary atherosclerosis; Aβ induces aortic valve interstitial cell osteogenesis, and then calcific aortic valve disease (CAVD) occurs. sAPP associates with p62/SQSTM1-ubiquitin to form inclusion bodies in cardiomyocytes and participates in basophilic degeneration.

APP is mainly produced by neurons, glial cells and blood vessels. APP is cleaved by the β-secretory enzyme BACE1 to produce Aβ. Aβ1-40 depositions are usually found in CAA, and Aβ1-42 is associated with the course of AD (Stakos et al., 2020). Aβ is not a unique product of APP decomposition. δ and η secreting enzymes can cleave APP to produce two prominent fragments, sAPPδ and sAPPη (Willem et al., 2015; Zhang et al., 2015). When the production and clearance of amyloid protein are out of balance, Aβ can be excessively deposited in the brain parenchyma or cerebrovascular, inducing neurodegeneration (Stakos et al., 2020). In addition, Aβ beyond the brain can also be deposited in myocardial cells and vascular endothelium (Figure 3).

##### 3.1.3.1 Aβ aggravates ischemic cardiac injury

Recent studies have revealed that Aβ aggregation in cardiac endothelial cells and cardiomyocytes may directly mediate cytotoxic injury and play a strong part in ischemic heart disease and cardiac reperfusion injury (Greco et al., 2017). Aβ can directly increase oxidative stress, promote cardiac endothelial cell apoptosis, and then cause vascular injury (Greco et al., 2017). The main mechanism of vascular injury is that Aβ accumulation in mitochondria weakens mitochondrial function and causes ROS production increased and mitochondrial oxidation capacity decreased (Daniele et al., 2020). Aβ promotes inflammatory response and the production of pro-inflammatory factors, which can cause endothelial cell apoptosis and increase vascular endothelial permeability (Bogner et al., 2014; Hellenthal et al., 2022). Injured endothelial cells can regulate BACE1 transcription by secreting hypoxia-inducing factor-1α (HIF-1α), nuclear factor (NF)-κB and GATA-1, then promote APP processing and Aβ production (Cole and Vassar, 2007). Abnormal BACE1 expression and activity promoted Aβ accumulation in cardiomyocytes and endothelial cells (Zhou et al., 2022). Circulating macrophages can phagocytose APP and increase local and circulating Aβ concentrations through BACE1 processing (Shigematsu et al., 1992). ApoE participates in the regulation of Aβ metabolism, and its isoform ApoE4 has low affinity with Aβ, which reduces the clearance of Aβ. The mismatch between Aβ and apolipoprotein E (ApoE) promotes coronary atherosclerosis and then ischemic cardiac disease (Kim et al., 2009; Greco et al., 2017). Aβ can up-regulate the expression of low-density lipoprotein receptor (LDLR) and low-density lipoprotein receptor-related protein (LRP1) on endothelial cell membrane, which bring about a large amount of lipid accumulation in coronary intima (Gagno et al., 2020). After endothelial cells impaired by lipid aggregation, Aβ can induce platelet aggregation and degranulation, further promoting atherosclerotic plaque progression or micro-thrombotic events, and finally cause coronary atherosclerosis, which accompanied by tissue and cellular ischemia and hypoxia (Mehta and Mehta, 1979; Gagno et al., 2020; Li and Liu, 2022).

Furtherly, local ischemia results in endothelial cell death, collagen exposure, even release of intracellular Aβ. The escaped Aβ can fix to the exposed collagen, causing more extracellular Aβ deposition (Gagno et al., 2020). Moreover, extracellular amyloid deposits aggravate inflammation and endothelial damage, promote thrombosis, and worsen myocardial hypoxia and dysfunction (Janelidze et al., 2016).

##### 3.1.3.2 Aβ promotes calcification of cardiac valves

Calcific aortic valve disease (CAVD) is a cardiovascular disease with a high incidence in developed countries, characterized by progressive fibrosis and calcified remodeling of the valve. Aortic valve interstitial cells (AVICs) are the main cell types in aortic valve leaflets, and they also engage in valve calcification. Inflammatory cell infiltration and osteoblast differentiation are the main pathological processes of aortic valve (AV) calcification (Alexopoulos et al., 2012).

Immunohistochemical staining showed that there were 2–3 times of Aβ and receptor for advanced glycation end products (RAGE) in calcified AV compared with normal AV (Wang et al., 2020). Interaction of RAGE to Aβ, advanced glycation end products (AGE)s, and S100B activates MEK, ERK1/2, p38, and the transcription factor NF-κB, which induces the release of pro-inflammatory cytokines (Jangde et al., 2020). RAGE can also bind to Aβ, causing ROS production, mitogen-activated protein kinases (MAPK) activation, and osteogenic differentiation of AVICs (Jangde et al., 2020; Wang et al., 2020). After stimulating human AVICs with different concentrations of Aβ, the expression levels of RAGE and osteogenic markers in AVICs increased significantly in a dose-dependent manner (Wang et al., 2020). RAGE-targeted siRNA can significantly inhibit the differentiation of AVICs into osteoblasts (Wang et al., 2020). Activation of RAGE pathway can up-regulate the expression of pro-inflammatory cytokines (interleukin (IL)-1, IL-6, monocyte chemoattractant protein-1 (MCP-1)) and osteogenic biomarkers (bone morphogenetic protein (BMP)2, runt-related transcription factor (Runx)2), induce endoplasmic reticulum stress, and then contribute to AVICs osteoblast differentiation and inflammation aggravation (Wang et al., 2017). In conclusion, the Aβ/RAGE pathway is involved in the progression of AV calcification. The deposit of Aβ in aortic valve can activate RAGE pathway, and the activation of RAGE transmits cell surface signals to intracellular pathways such as NF-κB, MAPK and ERK1/2, thus activating the downstream protein. This pathway aggravates inflammation and endoplasmic reticulum stress, promotes AVICs ossification, and ultimately CAVD occurs.

##### 3.1.3.3 sAPP causes basophilic degeneration of cardiomyocytes

Basophilic degeneration (BD) of the heart is a special kind of cardiomyopathy with characteristic inclusion bodies, namely P62/sequestosome (SQSTM)1-ubiquitin aggregates. P62/SQSTM1 is the hallmark of AD, Parkinson’s disease, and many other chronic degenerative diseases (Ma et al., 2019). BD is a special manifestation of myocardial cell aging and injury, which may be related to APP fragment and CAA (Krämer et al., 2018). Autopsy results of 62 BD cases showed that basophilic variant cardiomyocytes with periodic acid-Schiff reaction staining (PAS)-positive expressed p62/SQSTM1 when detected by anti-p62/SQSTM1 antibodies. PAS-positive APP fragments were co-located with p62/SQSTM1-ubiquitin (Krämer et al., 2018). Cytoplasmic inclusions in BD cardiomyocytes are composed of sAPPδ, sAPPη or APP short N- terminal fragments, which form inclusion bodies with p62/SQSTM1-ubiquitin. These inclusion bodies are related to the severity of CAA. From the anatomical distribution of the whole heart, PAS positive BD-inclusion bodies can be observed in both ventricular and atrial cardiomyocytes, with the highest content in atrium and the lowest content in ventricular septum (Krämer et al., 2018). Therefore, the accumulation of p62/SQSTM1-ubiquitin -BD inclusion bodies are associated with cardiomyocytes BD injury.

### 3.2 Mechanism of “second hit” of CAA on heart (post-stroke)

**FIGURE 4:**
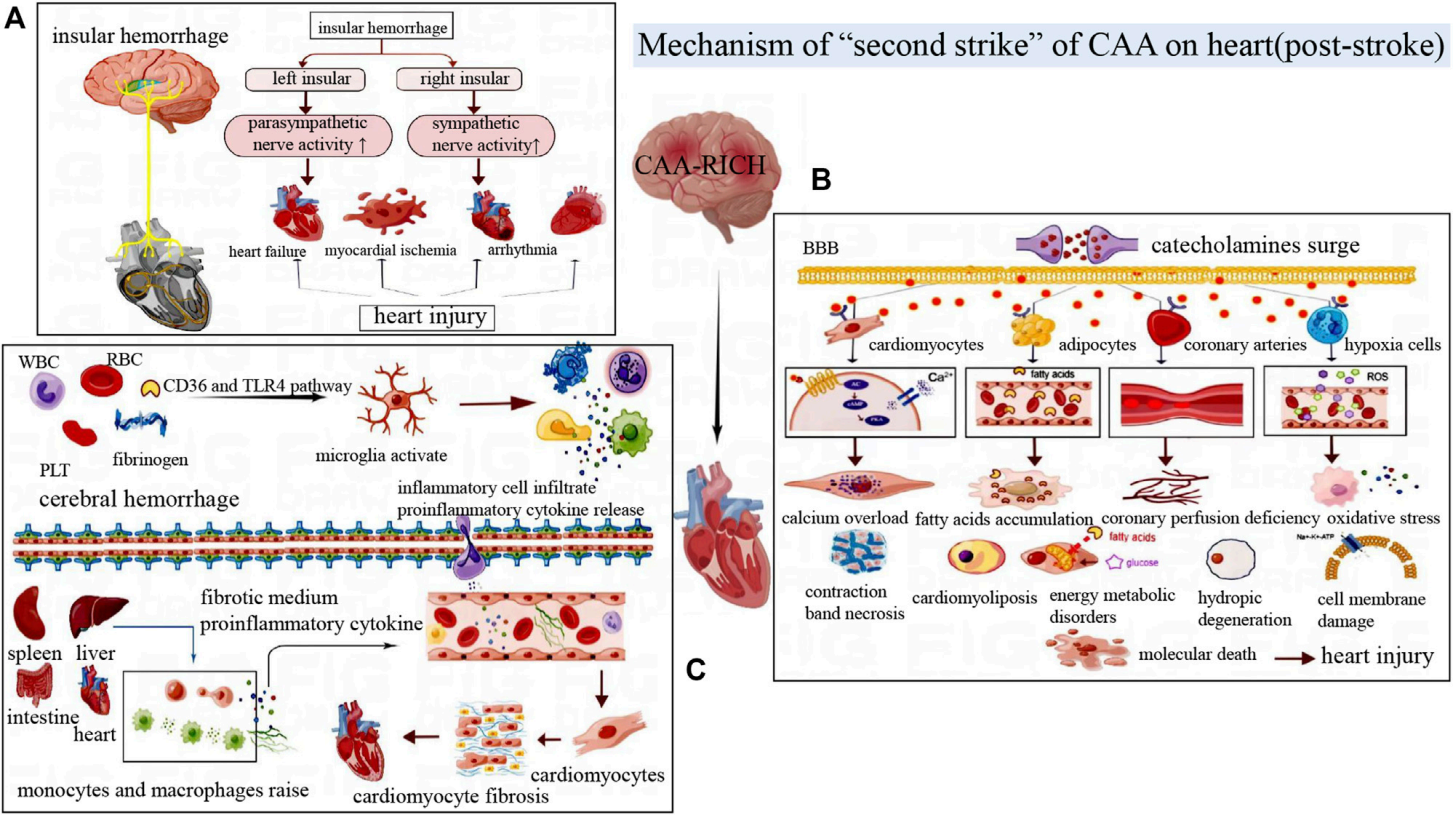
**(A)** The left and right insular cortexes have different lateralizing effects on the autonomic nervous system. Damage to the right insular cortex often activates the sympathetic nerve, while damage to the left insular cortex often activates the parasympathetic nerve, resulting in different cardiac injury manifestations. **(B)** Post-stroke catecholamines surge, which acts on cardiomyocytes, adipocytes, inflammatory cells and blood vessels, then leading to myocardial contraction band necrosis, myocardial steatosis, vasoconstriction myocardial ischemia, excessive oxidative stress and other manifestations of cardiac injury. **(C)** The release of blood components activates microglia, followed by the release of inflammatory factors and cytokines that lead to a systemic inflammatory response by damaging the blood-brain barrier. Mononuclear macrophage recruitment in the liver, spleen, and intestine aggravates cardiac inflammation and eventually leads to myocardial fibrosis and other damage.

With the widespread application of anticoagulant drugs and antiplatelet drugs in the elderly, the incidence of CAA-RICH is increasing (Béjot et al., 2013). According to statistics, 20%–40% of patients with cerebral hemorrhage have cardiac injuries, which are related to the severity of the disease severity, discharge disposition, and mortality (Alkhachroum et al., 2019). The clinical and pathological manifestations of post-stroke cardiac events are diverse, as shown in Table 1 (Sposato et al., 2020). In general, CAA-RICH brain injury can cause neurogenic heart damage or aggravate existing heart disease, and this secondary cardiac injury can be called “second hit” on heart (Figure 4).

**TABLE 1:** Clinical and pathological manifestations of Stroke-heart syndrome.

Pathophysiology		Clinical manifestations
Systemic	Focal	1. Ischemic and non-ischemic acute myocardial injury, characterized by elevated cardiac troponin (cTn) and no symptom
1. Inflammation	1. Inflammation	2. Acute myocardial infarction
2. Central autonomic nervous disorder	2. Sympathetic nerve sprouting	3. Ventricular dysfunction, heart failure and post-stroke Takotsubo syndrome
3. Catecholamine release	3. Cardiac structural changes	4. ECG changes and arrhythmia
4. Apoptosis signal	4. Vascular wall abnormalities	5. Neurogenic sudden cardiac death

#### 3.2.1 Impairment of brain-heart neural network system caused by CAA-RICH

The heart has its own internal conduction system, called intrinsic cardiac nervous system, which is mainly controlled by the central autonomic nerve network of the brain. And this system can regulate the heart rate and myocardial contractility. At the same time, the brain also receives negative feedback from the afferent impulses of cardiac muscle and cardiac baroreceptors (Bieber et al., 2017). Central autonomic neural network system is mainly distributed in the extensive network of cortex, subcortex and brainstem region. And it is connected to the endogenous cardiac nervous system through the exogenous cardiac nervous system composed of sympathetic and parasympathetic nerves, and then transmits the regulatory signals of the brain to the cardiovascular system (Lin et al., 2021).

Deep insular hemorrhage can disrupt the functional regulatory areas of the central autonomic nervous system. The insular cortex is an important part of the central autonomic nerve network that affects the heart (Nagai et al., 2010). It has been proved that the insular cortex has obvious laterality, that is, stimulation of the left insular cortex can cause parasympathetic excitation of the heart, while stimulation of the right insular cortex can cause sympathetic excitation (Oppenheimer and Cechetto, 2016). The right insular cortex injury caused excessive sympathetic activity and decreased parasympathetic activity (Krause et al., 2017). The lesion can also cause cardiomyocyte lysis and cardiomyocyte work enhancement, leading to relative ischemia and acute myocardial injury (Krause et al., 2017). Cerebrovascular injury in the adjacent right insular cortex is often highly correlated with acute myocardial injury after stroke (Nagai et al., 2010). Excessive sympathetic nerve activity caused by right insular injury can also increase heart rate variability and even induce arrhythmia (Oppenheimer, 1994). The left insular injury seems to be related to the increased risk of cardiac events such as myocardial infarction, angina pectoris and congestive heart failure (Cheshire and Saper, 2006). In short, central autonomic dysfunction can induce autonomic function disturbance, which is one of the important pathogenesis of cardiovascular injury after stroke.

#### 3.2.2 The catecholamine surge caused by CAA-RICH

Catecholamines include epinephrine, norepinephrine and dopamine, which are mainly secreted by the medulla of the adrenal glands. In the rat model of hematoma, the plasma noradrenaline level of cerebral hemorrhage began to increase at 0.5 h after hemorrhage, reached its peak at 24h, then gradually decreased at 48 h (Liang et al., 2014). Besides, serum CK-MB level began to increase at 6 h after cerebral hemorrhage, reached its peak at 24 h, and then decreased. Myocardial histopathological examination further confirmed that the induction and development of cerebral hemorrhage were involved in myocardial degeneration and necrosis (Liang et al., 2014).

Catecholamine surges are one of the possible mechanisms for brain-heart syndrome (Lin et al., 2021). After the organism suffers from acute stress, the sympathetic nerve releases impulse quickly, resulting in excessive activation of the hypothalamic-pituitary-adrenal axis and massive release of catecholamine (Kenigsberg et al., 2019). At the same time, the BBB is destroyed, catecholamines in the brain can diffuse into the blood circulation (Dilraj et al., 1992; Semenas et al., 2014). Plasma overloading catecholamine can cause cardiac injury by direct myocardial toxicity, aggravating myocardial ischemia and hypoxia, interfering with cardiomyocyte metabolism, and coronary microvascular dysfunction (Kenigsberg et al., 2019). Not only that, but circulating catecholamines can activate the adrenergic receptors of adipocytes, increasing the release of fatty acids. And aggregation of free fatty acids in myocardial cells causes energy metabolism disorder, inflammation and cardiotoxicity (Andreis and Singer, 2016). Catecholamine can activate the cascade reaction of G protein-adenylate cyclase (AC)-cyclic adenosine monophosphate (cAMP)- protein kinase A (PKA) in myocardial cells, generating calcium channel disorder, calcium overload in myocardial cells, myocardial fibrosis, contraction zone necrosis and other pathological changes (Caspi et al., 1998). In addition, high level catecholamines can cause coronary artery vasospasm, myocardial contraction and heart rate increasing, unbalance of myocardial oxygen supply and demand, which aggravate myocardial ischemia and hypoxia (Mann et al., 1992). After ischemia and hypoxia, oxygen radicals and catecholamine oxidation products are released, which can lead to cell membrane damage, mitochondrial dysfunction, myocardial cell damage and cardiovascular toxicity (Behonick et al., 2001). The increase of catecholamine level will also affect cerebrovascular contraction, promote inflammatory response, aggravate ischemia, hypoxia and swelling of brain tissue, and the vicious cycle will lead to increased heart damage (Min et al., 2009). However, due to CAA-induced LC amyloidosis with dysregulated of adrenergic receptor expression, catecholamine surge after CAA-RICH would further aggravate the cardiac injury (Gannon et al., 2015).

#### 3.2.3 The systemic inflammatory reaction caused by CAA-RICH

The primary brain injury of cerebral hemorrhage begins to occur in the first few hours, which is mainly due to the mechanical damage caused by hematoma formation. The large amount of bleeding and hematoma enlargement may bring about poor prognosis (Lin et al., 2021). However, there is growing evidence shows that the main cause of secondary injury in brain hemorrhage is the inflammatory response.

A prospective study of 200 consecutive stroke patients found that 60% developed the systemic inflammatory response within 15 days after the disease onset, and the number of people with systemic inflammation decreased over time (Kalita et al., 2015). Systemic inflammatory response is a complicated process, in which immune cells, pro-inflammatory cytokines, chemokines and sympathetic nerve activities are involved (Bone, 1996; Pereira and Leite, 2016). The basic processes include: microglia activation, release of cytokines and chemokines, infiltration of polymorphonuclear leukocytes and macrophages, activation of pro-inflammatory transcription factors, proliferation of astrocytes and expression of inflammation-related enzymes and so on (Aronowski and Hall, 2005). When cerebral hemorrhage first occurs, blood components such as erythrocyte, leukocytes, macrophages, hemoglobin and plasma proteins (thrombin and fibrinogen) enter the brain parenchyma to form hematoma. The blood components activate microglia through pathways such as CD36 or toll-like receptor (TLR)4, after then the inflammatory response subsequently initiated (Wang and Doré, 2007).

The resident microglia and astrocytes are rapidly activated as early inflammatory cells, accompanied by infiltration of circulating inflammatory cells such as leukocytes and macrophages (Wang and Doré, 2007). However, excessive activation of microglia and circulating inflammatory cells can produce various cytokines, chemokines, free radicals, nitric oxide and other toxic chemicals. These substances can induce a cascade of increased inflammation. For example, inflammatory cytokines and encephaledema can lead to tissue damage, BBB destruction and cerebral cell death (Zhou et al., 2014). A large number of inflammatory cells, inflammatory mediators and cytokines accumulate in the blood circulation after cerebral hemorrhage, which directly or indirectly damages myocardial cells (Chen et al., 2017). Tumor necrosis factor (TNF)-α can mediate the expression of inflammatory genes in myocardial cells, atherosclerosis of cardiac vessels, apoptosis of myocardial cells, and bad remodeling after myocardial infarction (Sagris et al., 2021; Al-Botaty et al., 2022). ILs is associated with acute myocarditis, myocardial fibrosis, dilated cardiomyopathy and heart failure (Bartekova et al., 2018). During inflammatory reaction activates, local proliferation of macrophages and monocytes recruitment occur in spleen, intestine, heart and other organs, which produce a large number of pro-inflammatory cytokines and inhibit cardiac remodeling (Hulsmans et al., 2016).

To sum up, the bleeding volume and hematoma size of cerebral hemorrhage are closely related to the prognosis. Cerebral hemorrhage not only directly damages the central nervous system through mechanical action, but also activates the inflammatory reaction to produce multiple brain and heart injuries.

### 3.3 The vicious circle of CAA-CI based on “brain-heart axis”

The formation of CAA-CI is not a simple loop that begins with CAA and ends with myocardial injury. The course of CAA-CI contains many vicious cycles based on the brain-heart axis. In order for researchers to have a deeper understanding of CAA-related myocardial injury, we review the vicious circle events formed in the course of brain-heart axis-related myocardial injury.

#### 3.3.1 Neuroendocrine-mediated vicious circle

The hypothalamic-pituitary-adrenal (HPA) axis is composed of three endocrine glands, the hypothalamus, the pituitary gland and the adrenal gland, which is an important part of the neuroendocrine system. The paraventricular nucleus of the hypothalamus is the core of the HPA axis, and it can secrete corticotropin-releasing hormone and antidiuretic hormone. During the cerebral hemorrhage, corticotropin-releasing hormone (CRH) promotes the secretion of adreno-cortico-tropic-hormone (ACTH) by the pituitary gland, which increases the level of cortisol in the blood (Whitnall, 1993). Long-term elevated cortisol is neurotoxic and may increase post-stroke mortality (Barugh et al., 2014).

The autonomic nervous system makes connections between brain and heart by secreting catecholamines. Norepinephrine is the main neurotransmitter of the sympathetic nerve. When cerebral hemorrhage occurs, norepinephrine secreted by sympathetic nerve surges, which can accelerate the heart rate and enhance cardiac contractility (Mertes et al., 1994; Díaz-Araya et al., 2015). Norepinephrine can also cause myocardial hypertrophy or myocardial ischemia, and eventually lead to insufficient blood supply to the brain, forming a vicious cycle (Samuels, 1987). The sympathetic nerve releases catecholamines directly, which can cause cardiotoxicity and may result in edema of the hypokinesia area, transient fibrosis, inflammation, and necrosis of the contractile zone (Nef et al., 2009).

The renin angiotensin system (RAS) regulates blood pressure and electrolyte balance, and angiotensin (Ang) II in RAS plays an important role in vasoconstriction, cell proliferation, inflammation, and apoptosis. Intracerebral injury activates the sympathetic nerve, and sympathetic hyperactivity leads to the activation of juxtaglomerular cells and increased release of renin and Ang II, contributing to the development of cardiovascular diseases such as atherosclerosis, hypertension and heart failure (Koba, 2018). These cardiac conditions can also further aggravate brain injury. Cardiogenic stroke accounts for 9% of all strokes, and heart failure influences stroke mainly through protein ubiquitination, Wnt signaling pathways, and exosomes (Pullicino and Homma, 2010; Liu et al., 2021).

#### 3.3.2 Inflammation-mediated vicious circle

When the sympathetic nerve is activated during cerebral hemorrhage, the sympathetic nerve endings of immune organs release Norepinephrine, which binds to adrenergic receptors on various cell populations. Inflammation is also closely related to cardiovascular disease (Ruparelia et al., 2017). When myocardial infarction occurs, the recruited macrophages will destabilize the plaque and promote thrombotic events (Dutta et al., 2012). Lymphocytes influx begins 48 h after acute ischemic stroke, and they promote a deleterious inflammatory cascade that causes delayed brain damage (Emsley et al., 2003). The spleen increases inflammation after stroke by contributing the production of lymphocytes and proinflammatory cytokines, which can cause peripheral inflammation activation (Offner et al., 2006). Elevated levels of TNF-α can induce degradation of troponin I, resulting in impaired cardiac contractility and aggravated cerebral ischemia (Adams et al., 2007).

After stroke, damaged brain cells release damage-associated molecular patterns (DAMP) (Shichita et al., 2012). DAMPs can promote the activation of microglia and astrocytes. Moreover, when brain injury occurs, it will promote the transformation of microglia cells from anti-inflammation (M2) to pro-inflammation (M1) and aggravate the inflammatory response of nerve tissue (Shi et al., 2019). Brain-derived antigens can enter the systemic circulation through the ruptured BBB (Marsh et al., 2009). Furthermore, the systemic inflammatory response can be activated by stroke, and it can bring about adverse outcomes of heart failure and cardiac disease through TLR4 and chronic inflammation (Mann, 2015; Chen et al., 2017). In general, brain injury creates a vicious cycle of inflammation in the brain. Inflammatory cytokines can spill out from the damaged BBB into the peripheral circulation and stimulate the spleen to increase circulating immune cells to participate in inflammation, thus causing systemic inflammatory response. This systemic inflammatory response can worsen cardiac injury and even cause heart failure. When the contraction of the heart is affected, it can aggravate cerebral ischemia and hypoxia.

#### 3.3.3 Special protein interaction-mediated vicious circle

Thioredoxin interacting protein (TXNIP) is a 46 kDa protein composed of 391 amino acid residues, and its gene is located on human chromosome 1q21.1. In the brains of AD patients, TXNIP contributes to oxidative damage, hyperphosphorylation of tau and other changes (Melone et al., 2018). TXNIP is associated with neurodegenerative diseases and promotes the progression of AD and Parkinson’s disease by activating the NOD-like receptor protein 3(NLRP3) inflammasome (Tsubaki et al., 2020). Epigenetic modification of TXNIP can increase the risk of cardiovascular disease (Richard et al., 2017). Circulating TXNIP are related to coronary artery disease and cardiac disease (Zhang et al., 2017). To sum up, TXNIP may be an important intermedium linked CAA with cardiovascular diseases.

In addition, studies have shown that heart failure and AD share similar genetic traits. In familial diseases, the two diseases have the same variation in the presenilin (PSEN)1 or PSEN2 gene. The same missense mutation in the PSEN1 and PSEN2 genes was found in AD and sporadic cases of iDCM, and new genetic variants in the gene promoter region affecting protein expression levels were explored (Li et al., 2006). Heart failure reduces blood flow to the brain, leading to a metabolic energy crisis. This energy shortage induces acidosis and oxidative stress in multiple brain regions, ultimately neuronal degeneration occurs (Moreira et al., 2005; Pirchl and Humpel, 2009). Damage to the brain is often accompanied by abnormal secretion of neurotransmitters into the bloodstream, which in turn affects the cardiac function.

#### 3.3.4 Abnormal metabolites-mediated vicious circle

Cofilin is a small-molecule actin binding protein that regulates intracellular actin depolymerization and maintains cell morphology, polarity and migration. Abnormal aggregation of this protein is often found in neurodegenerative diseases, myocardial ischemia and dilated cardiomyopathy (Gianni et al., 2010; Subramanian et al., 2015). Cofilin aggregates affect cardiac contractility, which in turn reduces the blood supply to the brain (Subramanian et al., 2015). They can also accumulate in the brain and cause neurodegeneration (Schönhofen et al., 2014). The accumulation of misfolded proteins such as Aβ, ATTR and cofilin can lead to diastolic dysfunction of the heart and then exacerbating brain damage in AD patients (Willis and Patterson, 2013).

Studies have found that patients with diabetic cardiomyopathy have an accumulation of amylin in the heart (Despa and Decarli, 2013). Amylin and plaques can also appear in the cerebrovascular tissues of AD patients with diabetes mellitus. In addition, amylin deposits were found in the cerebrovascular and parenchyma of late onset AD (Jackson et al., 2013). Amylin can form functional aggregates, which have been proved to link with oxidative stress, inflammation, and changes in the vascular system (Kawahara et al., 2000).

Aβ is a hallmark of CAA and AD. Aβ is generally deposited in the circulatory system at the onset of CAA, from the leptomeningeal and cortical cerebral microvessels to intracranial vessels, to the carotid artery, aorta or coronary artery wall and even the heart (Tublin et al., 2019). Under normal circumstances, there is a balance between the production and clearance of Aβ in the central nervous system and even the whole body (Mawuenyega et al., 2010). Breaking this balance may generate abnormal accumulation of Aβ1-40 in blood, blood vessel wall and heart tissue, and eventually bring about the occurrence or aggravation of cardiovascular diseases. Aβ1-40 is mainly accumulated in blood vessels, while Aβ1-42 is mainly related to cognitive impairment of AD (Stakos et al., 2020). Abnormal accumulation of Aβ can give rise to plaque rupture, thrombosis, and finally acute coronary syndrome (Chong et al., 2001). Cardiac dysfunction usually causes brain ischemia and hypoxia. In addition to Aβ, other types of amyloid are also associated with vascular amyloidosis (Revesz et al., 2002). In another neurodegenerative disease, Familial Danish Dementia (FDD), the intracranial blood vessels also have deposits of amyloid, called Danish amyloid (ADan) (Vidal et al., 2000b). In a mouse model of FDD, it was demonstrated that ADan could promote the hyperphosphorylation and misfolding of tau protein, resulting in tau aggregation and the vicious circle of neurovascular injury (You et al., 2019).

## 4 Diagnosis of CAA-CI: Biomarker, imaging or genetic test?

The clinical manifestations of CAA are non-specific, and histopathological diagnosis is the only gold standard. The diagnostic criteria of CAA in Boston proposed in 1990s suggested that CAA could be diagnosed by imaging without brain pathology (Greenberg and Vonsattel, 1997) (Table 2). In 2010, Linn et al. (2010) modified the diagnostic criteria, believing that adding superficial siderosis to the diagnostic criteria could improve the diagnostic sensitivity of CAA-related cerebral hemorrhage. On this basis, the Boston diagnostic criteria for CAA version 2.0 had been published in Lancet Neurology in August 2022. This 2.0 diagnosis criteria refined the imaging diagnosis of cerebral hemorrhage, added clinical manifestations other than cerebral hemorrhage and modified some values (Table 3). For instance, version 2.0 proposes to lower the initial age of onset from 55 to 50 years, and emphasizes the significance of excluding other diseases (Kozberg et al., 2021; Charidimou et al., 2022).

**TABLE 2:** Boston criteria version 1.0 for CAA (Greenberg and Vonsattel, 1997).

Diagnostic type	Improved CAA Boston diagnostic criteria
Definite CAA	Full brain post-mortem examination reveals
	1. Lobar, cortical, or subcortical hemorrhage
	2. Severe CAA
Probable CAA with supporting pathology	Clinical data and pathological tissue (evacuated hematoma or cortical biopsy) reveals
	1. Lobar, cortical, or subcortical hemorrhage
	2. Some degree of CAA.
Probable CAA	Clinical data and MRI result reveals
	1. Patients age≥55 years
	2. Multiple hemorrhages (located in the above area)
	3. Exclude other causes of hemorrhage
Possible CAA	Clinical data and MRI result reveals
	1. Patients age≥55years
	2. A single hemorrhage (located in the above area)
	3. Exclude other causes of hemorrhage

**TABLE 3:** Boston criteria version 2.0 for CAA.

Diagnostic type	Improved CAA Boston diagnostic criteria Charidimou et al. (2022)
Definite CAA	Full brain post-mortem examination reveals
	1. Spontaneous ICH, TFNE, cSAH, cognitive disorders or dementia
	2. Severe CAA
	3. Exclude other possible diseases
Probable CAA with supporting pathology	Clinical data and pathological tissue (evacuated hematoma or cortical biopsy) reveals
	1. Spontaneous ICH, TFNE, cSAH, cognitive disorders or dementia
	2. Some degree of CAA
	3. Exclude other possible diseases
Probable CAA	Age≥50 years, clinical data and MRI result reveals
	1. Spontaneous ICH, TFNE, cSAH, cognitive disorders or dementia
	2. Multiple lobar hemorrhagic lesions^a^
	OR
	1. One lobar hemorrhagic lesion and one white matter feature^a^ ^,^ ^b^
	2. Without any deep hemorrhagic lesions^c^
	3. Exclude other causes of hemorrhage^d^
Possible CAA	Age≥50 years, clinical data and MRI result reveals
	1.Spontaneous ICH, TFNE, cSAH, cognitive disorders or dementia
	2. A single lobar hemorrhagic lesion^a^
	OR
	1. One white matter feature^b^
	2. Without any deep hemorrhagic lesions^c^
	3. Exclude other causes of hemorrhage^d^

**Abbreviations:** ICH, intracerebral hemorrhage; TFNE, transient focal neurological episodes; cSAH, convexity subarachnoid hemorrhage.

^a^ **Description:**Lobar hemorrhagic lesions: intracerebral hemorrhage, cerebral microbleeds, or foci of cortical superficial siderosis or cSAH.

^b^ White mater feature: severe perivascular spaces in the centrum semiovale or white matter hyperintensities in a multispot pattern.

^c^ Hemorrhaic lesion in cerebellum not counted as either lobar or deep hemorrhagic lesion.

^d^ Other cases of hemorrhagic lesions: antecedent head trauma, hemorrhagic transformation of an ischemic stroke, arteriovenous malformation, hemorrhagic tumor, CNS, vasculitis. Other causes of cortical superficial siderosis and cSAH, should also be excluded.

Currently advanced diagnostic methods include amyloid imaging marker-florbetapir-PET and detection of ApoE genotypes, plasma Aβ, cerebrospinal fluid Aβ and tau concentrations, which have received much attention. ApoE 4 allele can contribute to atherosclerosis, and it will increase the prevalence of cardiovascular and cerebrovascular diseases and significantly promote vascular disease progression in patients with age. What’s more, it increases the genetic risk of AD and causes vascular diseases (Gottesman et al., 2017). In addition, researchers found that higher plasma Aβ level can be regarded as a predictor of Chronic coronary syndrome (CCS) development, it is also a sign of poor prognosis of CCS in CAA patients. Patients with CAA have decreased levels of Aβ and increased tau in CSF, which can be used as one of the auxiliary diagnoses of CAA (Weber et al., 2018).

Florbetapir-PET is a novel imaging method, which can also work in the diagnosis of possible CAA without clinical manifestations and CAA-CI. Studies have shown that florbetapir-PET has relatively high sensitivity and specificity in screening patients with normal cognition who may have CAA. The ARIC-PET study of the relationship between atrial heart disease, left ventricular structure function and brain amyloid protein confirmed that florbetapir standard uptake value ratio (SUVR) elevation would significantly change left atrial volume index and left ventricular structure echocardiography (Johansen et al., 2019; Johansen et al., 2020). However, until now, further definite diagnosis still depends on reliable histopathological results, and more feasible and convenient diagnostic methods need to be explored.

## 5 Therapy of CAA-CI

So far, there is no specific and effective treatment for CAA. The research on new therapeutic strategies to prevent or delay the progression of CAA still needs great advances. Clinically, the commonly used methods for the treatment and prevention of CAA complications include lifestyle changes, continuous blood pressure management, strictly control of anticoagulation or antiplatelet indications, and early use of statin drugs. But there is no conclusive evidence that these methods have a significant effect on reducing the plasma concentration of Aβ (Stakos et al., 2020). Novel drug candidate of CAA, anti-amyloid antibody, mediates passive immunotherapy and accelerates the clearance of accumulated or soluble Aβ (Piazza et al., 2013). However, effectiveness of that drug is inconsistent, and some studies have even found meningoencephalitis in some subjects who received the passive immunotherapy (Kozberg et al., 2021).

Enhancing vascular motility is another non-pharmaceutical therapy for CAA. It can clear Aβ by speeding up perivascular drainage. The efficacy of promoting healthy sleep and non-invasive sensory stimulation to enhance vascular motility and increase Aβ clearance needs to be further evaluated (Fultz et al., 2019). In addition, when CAA patients have cerebral hemorrhage and other stroke events, removing hematoma, controlling the amount of bleeding, reducing intracranial pressure and preventing brain edema as soon as possible would be the key for therapy. Controlling inflammatory response and sympathetic nerve activity and reducing oxidative stress are also essential for CAA-RICH alleviation. The therapy of CAA and its associated complications is a great challenge for brain surgeons and intensive care physicians. Other treatments still need to be explored around the pathogenesis and different stages of the disease.

## 6 Conclusion and prospection

When CAA occurs, amyloid substances are deposited in the walls of small and medium blood vessels of the cerebral cortex and meninges (Stakos et al., 2020). The aggregation not only impairs the brain tissue, but also causes the damage of heart, kidney, intestine and other organs, with brain-heart syndrome being the most prominent manifestation. CAA is the second leading cause of cerebral hemorrhage, and the occurrence of bleeding events can further aggravate CAA-CI. When proteins misfolding, clumping and depositing in the cardiomyocytes, they will affect the pumping function of heart and eventually lead to CA. Aβ, tau and ATTR are the key proteins that bridge AD/CAA and CA. From the pathophysiological perspective of CAA-CI, there are “first hit” and “second hit” two phases. The formation of CAA-CI is also not a simple loop that starts with CAA and ends with myocardial injury, but actually contains multiple vicious cycles based on the brain-heart axis in the course of the disease.

So far, great progress has been made in CAA. In the therapy of CAA and its related complications, humanin may relieve memory impairment caused by Aβ, and gastrodin may be used to relieve CAA-related inflammation (Tajima et al., 2005; Dai et al., 2011). At present, the advanced therapeutic agents of CAA are anti-Aβ antibodies, including drug NC-758 and drug PF-04360,365. The drugs have been in clinical trials and is also used to treat AD (La Porte et al., 2012). In the future, AD and CAA should have their own drugs. This treatment will allow for one-to-one precision treatment of CAA and its resulting complications. The diagnosis of CAA involves many detection methods, mainly including imaging and biochemical detection. 7T MRI has a strong sensitivity for detecting microbleeds in the brain, but it may be replaced in the future by automated microbleeds detection systems, which have the advantages of saving time, repeatability and reliability (Koschmieder et al., 2022). Unlike CT and MRI, PET imaging can provide direct evidence of amyloid deposition in the blood vessels. It may in the future be a means of CAA early risk stratification and diagnosis of asymptomatic CAA. We believe that the onset age on CAA diagnosis in the future will tend to be younger. In addition, the importance of imaging will become more and more prominent, with higher requirements for precision and refinement. The clinical diagnosis and treatment measures are still limited, and the rate of misdiagnosis and missed diagnosis is still high. The clinicians often fail to make timely diagnosis or even fail to diagnose brain-heart syndrome in CAA. Therefore, it is urgent to further understand the pathogenesis of CAA-related brain-heart syndrome and help clinical medical staff to implement symptomatic and effective treatment. The complications of this disease and corresponding treatment plan need to be further studied and discussed.

## References

[B1] Adams V. Linke A. Wisloff U. Doring C. Erbs S. Krankel N. (2007). Myocardial expression of Murf-1 and MAFbx after induction of chronic heart failure: Effect on myocardial contractility. Cardiovasc Res. 73 (1), 120–129. 10.1016/j.cardiores.2006.10.026 17145048

[B2] Al-Botaty B. M. Elkhoely A. K El-Sayed E. Ahmed A. A. E. (2022). Ethyl pyruvate attenuates isoproterenol-induced myocardial infarction in rats: Insight to TNF-α-mediated apoptotic and necroptotic signaling interplay. Int. Immunopharmacol. 103, 108495. 10.1016/j.intimp.2021.108495 34973531

[B3] Alexopoulos A. Kaoukis A. Papadaki H. Pyrgakis V. (2012). Pathophysiologic mechanisms of calcific aortic stenosis. Ther. Adv. Cardiovasc Dis. 6 (2), 71–80. 10.1177/1753944712439337 22361851

[B4] Alkhachroum A. M. Miller B. Chami T. Tatsuoka C. Sila C. (2019). A troponin study on patients with ischemic stroke, intracerebral hemorrhage and subarachnoid hemorrhage: Type II myocardial infarction is significantly associated with stroke severity, discharge disposition and mortality. J. Clin. Neurosci. 64, 83–88. 10.1016/j.jocn.2019.04.005 31014907

[B5] Andreis D. T. Singer M. (2016). Catecholamines for inflammatory shock: A jekyll-and-hyde conundrum. Intensive Care Med. 42 (9), 1387–1397. 10.1007/s00134-016-4249-z 26873833

[B6] Andrews-Zwilling Y. Bien-Ly N. Xu Q. Li G. Bernardo A. Yoon S. Y. (2010). Apolipoprotein E4 causes age- and Tau-dependent impairment of GABAergic interneurons, leading to learning and memory deficits in mice. J. Neurosci. 30 (41), 13707–13717. 10.1523/jneurosci.4040-10.2010 20943911 PMC2988475

[B7] Aronowski J. Hall C. E. (2005). New horizons for primary intracerebral hemorrhage treatment: Experience from preclinical studies. Neurol. Res. 27 (3), 268–279. 10.1179/016164105x25225 15845210

[B8] Arvanitakis Z. Leurgans S. E. Wang Z. Wilson R. S. Bennett D. A. Schneider J. A. (2011). Cerebral amyloid angiopathy pathology and cognitive domains in older persons. Ann. Neurol. 69 (2), 320–327. 10.1002/ana.22112 21387377 PMC3228518

[B9] Askanas V. Engel W. K. Bilak M. Alvarez R. B. Selkoe D. J. (1994). Twisted tubulofilaments of inclusion body myositis muscle resemble paired helical filaments of Alzheimer brain and contain hyperphosphorylated tau. Am. J. Pathol. 144 (1), 177–187. 8291607 PMC1887131

[B10] Bartekova M. Radosinska J. Jelemensky M. Dhalla N. S. (2018). Role of cytokines and inflammation in heart function during health and disease. Heart Fail Rev. 23 (5), 733–758. 10.1007/s10741-018-9716-x 29862462

[B11] Barugh A. J. Gray P. Shenkin S. D. MacLullich A. M. J. Mead G. E. (2014). Cortisol levels and the severity and outcomes of acute stroke: A systematic review. J. Neurol. 261 (3), 533–545. 10.1007/s00415-013-7231-5 24477489 PMC4928702

[B12] Bayes-Genis A. Barallat J. de Antonio M. Domingo M. Zamora E. Vila J. (2017). Bloodstream amyloid-beta (1-40) peptide, cognition, and outcomes in heart failure. Rev. Esp. Cardiol. Engl. Ed. 70 (11), 924–932. 10.1016/j.rec.2017.02.021 28279654

[B13] Behonick G. S. Novak M. J. Nealley E. W. Baskin S. I. (2001). Toxicology update: The cardiotoxicity of the oxidative stress metabolites of catecholamines (aminochromes). J. Appl. Toxicol. 21 (1), S15–S22. 10.1002/jat.793 11920915

[B14] Béjot Y. Cordonnier C. Durier J. Aboa-Eboule C. Rouaud O. Giroud M. (2013). Intracerebral haemorrhage profiles are changing: Results from the dijon population-based study. Brain 136 (2), 658–664. 10.1093/brain/aws349 23378220

[B15] Bieber M. Werner R. A. Tanai E. Hofmann U. Higuchi T. Schuh K. (2017). Stroke-induced chronic systolic dysfunction driven by sympathetic overactivity. Ann. Neurol. 82 (5), 729–743. 10.1002/ana.25073 29023958 PMC5765487

[B16] Blair L. J. Frauen H. D. Zhang B. Nordhues B. A. Bijan S. Lin Y. C. (2015). Tau depletion prevents progressive blood-brain barrier damage in a mouse model of tauopathy. Acta Neuropathol. Commun. 3, 8. 10.1186/s40478-015-0186-2 25775028 PMC4353464

[B17] Bogner S. Bernreuther C. Matschke J. Barrera-Ocampo A. Sepulveda-Falla D. Leypoldt F. (2014). Immune activation in amyloid-β-related angiitis correlates with decreased parenchymal amyloid-β plaque load. Neurodegener. Dis. 13 (1), 38–44. 10.1159/000352020 24021982

[B18] Bone R. C. (1996). Toward a theory regarding the pathogenesis of the systemic inflammatory response syndrome: What we do and do not know about cytokine regulation. Crit. Care Med. 24 (1), 163–172. 10.1097/00003246-199601000-00026 8565523

[B19] Buxbaum J. N. Ye Z. Reixach N. Friske L. Levy C. Das P. (2008). Transthyretin protects Alzheimer's mice from the behavioral and biochemical effects of Abeta toxicity. Proc. Natl. Acad. Sci. U. S. A. 105 (7), 2681–2686. 10.1073/pnas.0712197105 18272491 PMC2268196

[B20] Caspi J. Coles J. G. Benson L. N. Wilson G. J. (1998). Brain damage and myocardial dysfunction: Protective effects of magnesium in the newborn pig. Ann. Thorac. Surg. 65 (6), 1730–1736. 10.1016/s0003-4975(98)00196-9 9647090

[B21] Cavallaro T. Klunk W. (2016). PiB-PET detects transthyretin-related cerebral amyloid angiopathy. Neurology 87 (8), 750–751. 10.1212/wnl.0000000000003018 27466476

[B22] Cermakova P. Eriksdotter M. Lund L. H. Winblad B. Religa P. Religa D. (2015). Heart failure and Alzheimer's disease. J. Intern Med. 277 (4), 406–425. 10.1111/joim.12287 25041352 PMC4409079

[B23] Charidimou A. Boulouis G. Frosch M. P. Baron J. C. Pasi M. Albucher J. F. (2022). The Boston criteria version 2.0 for cerebral amyloid angiopathy: A multicentre, retrospective, MRI-neuropathology diagnostic accuracy study. Lancet Neurol. 21 (8), 714–725. 10.1016/s1474-4422(22)00208-3 35841910 PMC9389452

[B24] Chen Z. Venkat P. Seyfried D. Chopp M. Yan T. Chen J. (2017). Brain-heart interaction: Cardiac complications after stroke. Circ. Res. 121 (4), 451–468. 10.1161/circresaha.117.311170 28775014 PMC5553569

[B25] Cheshire W. P. Jr. Saper C. B. (2006). The insular cortex and cardiac response to stroke. Neurology 66 (9), 1296–1297. 10.1212/01.wnl.0000219563.87204.7d 16682656

[B26] Chhatre S. Weiner M. G. Jayadevappa R. Johnson J. C. (2009). Incremental burden of congestive heart failure among elderly with Alzheimer's. Aging Ment. Health 13 (4), 628–634. 10.1080/13607860902774469 19629789

[B27] Chong Y. H. Sung J. H. Shin S. A. Chung J. H. Suh Y. H. (2001). Effects of the beta-amyloid and carboxyl-terminal fragment of Alzheimer's amyloid precursor protein on the production of the tumor necrosis factor-alpha and matrix metalloproteinase-9 by human monocytic THP-1. J. Biol. Chem. 276 (26), 23511–23517. 10.1074/jbc.M009466200 11306564

[B28] Cisternas P. Taylor X. Lasagna-Reeves C. A. (2019). The amyloid-tau-neuroinflammation Axis in the context of cerebral amyloid angiopathy. Int. J. Mol. Sci. 20 (24), 6319. 10.3390/ijms20246319 31847365 PMC6941131

[B29] Cole S. L. Vassar R. (2007). The Alzheimer's disease beta-secretase enzyme, BACE1. Mol. Neurodegener. 2, 22. 10.1186/1750-1326-2-22 18005427 PMC2211305

[B30] Dai J. N. Zong Y. Zhong L. M. Li Y. M. Zhang W. Bian L. G. (2011). Gastrodin inhibits expression of inducible NO synthase, cyclooxygenase-2 and proinflammatory cytokines in cultured LPS-stimulated microglia via MAPK pathways. PLoS One 6 (7), e21891. 10.1371/journal.pone.0021891 21765922 PMC3134470

[B31] Daniele G. DiLucia S. Masci P. G. Del Monte F. (2020). Heart and brain: Complex relationships for left ventricular dysfunction. Curr. Cardiol. Rep. 22 (8), 72. 10.1007/s11886-020-01318-w 32577917 PMC7309683

[B32] Debette S. Bauters C. Leys D. Lamblin N. Pasquier F. de Groote P. (2007). Prevalence and determinants of cognitive impairment in chronic heart failure patients. Congest. Heart Fail 13 (4), 205–208. 10.1111/j.1527-5299.2007.06612.x 17673872

[B33] Despa F. Decarli C. (2013). Amylin: What might be its role in Alzheimer's disease and how could this affect therapy? Expert Rev. Proteomics 10 (5), 403–405. 10.1586/14789450.2013.841549 24117198 PMC4068803

[B34] Díaz-Araya G. Vivar R. Humeres C. Boza P. Bolivar S. Munoz C. (2015). Cardiac fibroblasts as sentinel cells in cardiac tissue: Receptors, signaling pathways and cellular functions. Pharmacol. Res. 101, 30–40. 10.1016/j.phrs.2015.07.001 26151416

[B35] Dilraj A. Botha J. H. Rambiritch V. Miller R. van Dellen J. R. (1992). Levels of catecholamine in plasma and cerebrospinal fluid in aneurysmal subarachnoid hemorrhage. Neurosurgery 31 (1), 42–50. 10.1227/00006123-199207000-00007 1641109

[B36] Dutta P. Courties G. Wei Y. Leuschner F. Gorbatov R. Robbins C. S. (2012). Myocardial infarction accelerates atherosclerosis. Nature 487 (7407), 325–329. 10.1038/nature11260 22763456 PMC3401326

[B37] Emsley H. C. Smith C. J. Gavin C. M. Georgiou R. F. Vail A. Barberan E. M. (2003). An early and sustained peripheral inflammatory response in acute ischaemic stroke: Relationships with infection and atherosclerosis. J. Neuroimmunol. 139 (1-2), 93–101. 10.1016/s0165-5728(03)00134-6 12799026

[B38] Fidziańska A. Walczak E. Bekta P. Chojnowska L. (2011). Are cardiomyocytes able to generate pre-amyloid peptides? Folia Neuropathol. 49 (1), 64–70. 21455845

[B39] Fultz N. E. Bonmassar G. Setsompop K. Stickgold R. A. Rosen B. R. Polimeni J. R. (2019). Coupled electrophysiological, hemodynamic, and cerebrospinal fluid oscillations in human sleep. Science 366 (6465), 628–631. 10.1126/science.aax5440 31672896 PMC7309589

[B40] Gagno G. Ferro F. Fluca A. L. Janjusevic M. Rossi M. Sinagra G. (2020). From brain to heart: Possible role of amyloid-β in ischemic heart disease and ischemia-reperfusion injury. Int. J. Mol. Sci. 21 (24), 9655. 10.3390/ijms21249655 33348925 PMC7766370

[B41] Gamblin T. C. Chen F. Zambrano A. Abraha A. Lagalwar S. Guillozet A. L. (2003). Caspase cleavage of tau: Linking amyloid and neurofibrillary tangles in Alzheimer's disease. Proc. Natl. Acad. Sci. U. S. A. 100 (17), 10032–10037. 10.1073/pnas.1630428100 12888622 PMC187753

[B42] Gannon M. Che P. Chen Y. Jiao K. Roberson E. D. Wang Q. (2015). Noradrenergic dysfunction in Alzheimer's disease. Front. Neurosci. 9, 220. 10.3389/fnins.2015.00220 26136654 PMC4469831

[B43] Ghetti B. Piccardo P. Spillantini M. G. Ichimiya Y. Porro M. Perini F. (1996). Vascular variant of prion protein cerebral amyloidosis with tau-positive neurofibrillary tangles: The phenotype of the stop codon 145 mutation in PRNP. Proc. Natl. Acad. Sci. U. S. A. 93 (2), 744–748. 10.1073/pnas.93.2.744 8570627 PMC40125

[B44] Gianni D. Li A. Tesco G. McKay K. M. Moore J. Raygor K. (2010). Protein aggregates and novel presenilin gene variants in idiopathic dilated cardiomyopathy. Circulation 121 (10), 1216–1226. 10.1161/circulationaha.109.879510 20194882 PMC2844798

[B45] Godemann R. Biernat J. Mandelkow E. Mandelkow E. M. (1999). Phosphorylation of tau protein by recombinant GSK-3beta: Pronounced phosphorylation at select ser/thr-pro motifs but no phosphorylation at Ser262 in the repeat domain. FEBS Lett. 454 (1-2), 157–164. 10.1016/s0014-5793(99)00741-3 10413115

[B46] González-López E. Gallego-Delgado M. Guzzo-Merello G. de Haro-Del Moral F. J. Cobo-Marcos M. Robles C. (2015). Wild-type transthyretin amyloidosis as a cause of heart failure with preserved ejection fraction. Eur. Heart J. 36 (38), 2585–2594. 10.1093/eurheartj/ehv338 26224076

[B47] Gottesman R. F. Schneider A. L. C. Zhou Y. Coresh J. Green E. Gupta N. (2017). Association between midlife vascular risk factors and estimated brain amyloid deposition. Jama 317 (14), 1443–1450. 10.1001/jama.2017.3090 28399252 PMC5921896

[B48] Greco S. Zaccagnini G. Fuschi P. Voellenkle C. Carrara M. Sadeghi I. (2017). Increased BACE1-AS long noncoding RNA and β-amyloid levels in heart failure. Cardiovasc Res. 113 (5), 453–463. 10.1093/cvr/cvx013 28158647

[B49] Greenberg S. M. Vonsattel J. P. (1997). Diagnosis of cerebral amyloid angiopathy. Sensitivity and specificity of cortical biopsy. Stroke 28 (7), 1418–1422. 10.1161/01.str.28.7.1418 9227694

[B50] Hamasaki H. Shijo M. Nakamura A. Honda H. Yamada Y. Oda Y. (2022). Concurrent cardiac transthyretin and brain β amyloid accumulation among the older adults: The Hisayama study. Brain Pathol. 32 (1), e13014. 10.1111/bpa.13014 34390072 PMC8713523

[B51] Hellenthal K. E. M. Brabenec L. Wagner N. M. (2022). Regulation and dysregulation of endothelial permeability during systemic inflammation. Cells 11 (12), 1935. 10.3390/cells11121935 35741064 PMC9221661

[B52] Hernandez P. Lee G. Sjoberg M. Maccioni R. B. (2009). Tau phosphorylation by cdk5 and fyn in response to amyloid peptide abeta (25-35): Involvement of lipid rafts. J. Alzheimers Dis. 16 (1), 149–156. 10.3233/jad-2009-0933 19158430

[B53] Howlett G. J. Ryan T. M. Griffin M. D. W. (2019). Lipid-apolipoprotein interactions in amyloid fibril formation and relevance to atherosclerosis. Biochim. Biophys. Acta Proteins Proteom 1867 (5), 502–507. 10.1016/j.bbapap.2018.08.010 35818279

[B54] Hulsmans M. Sam F. Nahrendorf M. (2016). Monocyte and macrophage contributions to cardiac remodeling. J. Mol. Cell Cardiol. 93, 149–155. 10.1016/j.yjmcc.2015.11.015 26593722 PMC4846552

[B55] Iijima K. Gatt A. Iijima-Ando K. (2010). Tau Ser262 phosphorylation is critical for Abeta42-induced tau toxicity in a transgenic Drosophila model of Alzheimer's disease. Hum. Mol. Genet. 19 (15), 2947–2957. 10.1093/hmg/ddq200 20466736 PMC2901137

[B56] Jackson K. Barisone G. A. Diaz E. Jin L. w. DeCarli C. Despa F. (2013). Amylin deposition in the brain: A second amyloid in alzheimer disease? Ann. Neurol. 74 (4), 517–526. 10.1002/ana.23956 23794448 PMC3818462

[B57] Janelidze S. Stomrud E. Palmqvist S. Zetterberg H. van Westen D. Jeromin A. (2016). Plasma β-amyloid in Alzheimer's disease and vascular disease. Sci. Rep. 6, 26801. 10.1038/srep26801 27241045 PMC4886210

[B58] Jangde N. Ray R. Rai V. (2020). RAGE and its ligands: From pathogenesis to therapeutics. Crit. Rev. Biochem. Mol. Biol. 55 (6), 555–575. 10.1080/10409238.2020.1819194 32933340

[B59] Jia Y. Li G. Song G. Ye X. Yang Y. Lu K. (2022). SMASH-U aetiological classification: A predictor of long-term functional outcome after intracerebral haemorrhage. Eur. J. Neurol. 29 (1), 178–187. 10.1111/ene.15111 34534389

[B60] Johansen M. C. Mosley T. H. Knopman D. S. Wong D. F. Ndumele C. Shah A. M. (2020). Associations between atrial cardiopathy and cerebral amyloid: The ARIC-PET study. J. Am. Heart Assoc. 9 (24), e018399. 10.1161/jaha.120.018399 33289449 PMC7955392

[B61] Johansen M. C. Mosley T. H. Knopman D. S. Wong D. F. Wagenknecht L. E. Shah A. M. (2019). Associations between left ventricular structure, function, and cerebral amyloid: The ARIC-PET study. Stroke 50 (12), 3622–3624. 10.1161/strokeaha.119.027220 31597548 PMC6878182

[B62] Kalita J. Bastia J. Bhoi S. K. Misra U. K. (2015). Systemic inflammatory response syndrome predicts severity of stroke and outcome. J. Stroke Cerebrovasc. Dis. 24 (7), 1640–1648. 10.1016/j.jstrokecerebrovasdis.2015.03.057 25959500

[B63] Kang D. E. Roh S. E. Woo J. A. Liu T. Bu J. H. Jung A. R. (2011). The interface between cytoskeletal aberrations and mitochondrial dysfunction in Alzheimer's disease and related disorders. Exp. Neurobiol. 20 (2), 67–80. 10.5607/en.2011.20.2.67 22110363 PMC3213703

[B64] Kang H. Li X. Xiong K. Song Z. Tian J. Wen Y. (2021). The entry and egress of monocytes in atherosclerosis: A biochemical and biomechanical driven process. Cardiovasc Ther. 2021, 6642927. 10.1155/2021/6642927 34345249 PMC8282391

[B65] Kapasi A. Leurgans S. E. Arvanitakis Z. Barnes L. L. Bennett D. A. Schneider J. A. (2021). Aβ (amyloid beta) and tau tangle pathology modifies the association between small vessel disease and cortical microinfarcts. Stroke 52 (3), 1012–1021. 10.1161/strokeaha.120.031073 33567873 PMC7902459

[B66] Kawahara M. Kuroda Y. Arispe N. Rojas E. (2000). Alzheimer's beta-amyloid, human islet amylin, and prion protein fragment evoke intracellular free calcium elevations by a common mechanism in a hypothalamic GnRH neuronal cell line. J. Biol. Chem. 275 (19), 14077–14083. 10.1074/jbc.275.19.14077 10799482

[B67] Kenigsberg B. B. Barnett C. F. Mai J. C. Chang J. J. (2019). Neurogenic stunned myocardium in severe neurological injury. Curr. Neurol. Neurosci. Rep. 19 (11), 90. 10.1007/s11910-019-0999-7 31720870

[B68] Kim H. J. Park S. Cho H. Jang Y. K. San Lee J. Jang H. (2018). Assessment of extent and role of tau in subcortical vascular cognitive impairment using 18F-AV1451 positron emission tomography imaging. JAMA Neurol. 75 (8), 999–1007. 10.1001/jamaneurol.2018.0975 29799981 PMC6142932

[B69] Kim J. Basak J. M. Holtzman D. M. (2009). The role of apolipoprotein E in Alzheimer's disease. Neuron 63 (3), 287–303. 10.1016/j.neuron.2009.06.026 19679070 PMC3044446

[B70] Koba S. (2018). Angiotensin II, oxidative stress, and sympathetic nervous system hyperactivity in heart failure. Yonago Acta Med. 61 (2), 103–109. 10.33160/yam.2018.06.002 29946216 PMC6015797

[B71] Koschmieder K. Paul M. M. van den Heuvel T. L. A. van der Eerden A. W. van Ginneken B. Manniesing R. (2022). Automated detection of cerebral microbleeds via segmentation in susceptibility-weighted images of patients with traumatic brain injury. Neuroimage Clin. 35, 103027. 10.1016/j.nicl.2022.103027 35597029 PMC9127224

[B72] Kozberg M. G. Perosa V. Gurol M. E. van Veluw S. J. (2021). A practical approach to the management of cerebral amyloid angiopathy. Int. J. Stroke 16 (4), 356–369. 10.1177/1747493020974464 33252026 PMC9097498

[B73] Krämer L. M. Brettschneider J. Lennerz J. K. Walcher D. Fang L. Rosenbohm A. (2018). Amyloid precursor protein-fragments-containing inclusions in cardiomyocytes with basophilic degeneration and its association with cerebral amyloid angiopathy and myocardial fibrosis. Sci. Rep. 8 (1), 16594. 10.1038/s41598-018-34808-7 30413735 PMC6226444

[B74] Krause T. Werner K. Fiebach J. B. Villringer K. Piper S. K. Haeusler K. G. (2017). Stroke in right dorsal anterior insular cortex Is related to myocardial injury. Ann. Neurol. 81 (4), 502–511. 10.1002/ana.24906 28253544

[B75] Kuo Y. M. Kokjohn T. A. Watson M. D. Woods A. S. Cotter R. J. Sue L. I. (2000). Elevated abeta42 in skeletal muscle of Alzheimer disease patients suggests peripheral alterations of AbetaPP metabolism. Am. J. Pathol. 156 (3), 797–805. 10.1016/s0002-9440(10)64947-4 10702395 PMC1876838

[B76] La Porte S. L. Bollini S. S. Lanz T. A. Abdiche Y. N. Rusnak A. S. Ho W. H. (2012). Structural basis of C-terminal β-amyloid peptide binding by the antibody ponezumab for the treatment of Alzheimer's disease. J. Mol. Biol. 421 (4-5), 525–536. 10.1016/j.jmb.2011.11.047 22197375

[B77] Lamb Y. N. (2021). Tafamidis: A review in transthyretin amyloid cardiomyopathy. Am. J. Cardiovasc Drugs 21 (1), 113–121. 10.1007/s40256-020-00461-7 33469827

[B78] Leech T. Apaijai N. Palee S. Higgins L. A. Maneechote C. Chattipakorn N. (2020). Acute administration of metformin prior to cardiac ischemia/reperfusion injury protects brain injury. Eur. J. Pharmacol. 885, 173418. 10.1016/j.ejphar.2020.173418 32750367

[B79] Li D. Parks S. B. Kushner J. D. Nauman D. Burgess D. Ludwigsen S. (2006). Mutations of presenilin genes in dilated cardiomyopathy and heart failure. Am. J. Hum. Genet. 79 (6), 1030–1039. 10.1086/509900 17186461 PMC1698711

[B80] Li T. R. Liu F. Q. (2022). β-Amyloid promotes platelet activation and activated platelets act as bridge between risk factors and Alzheimer's disease. Mech. Ageing Dev. 207, 111725. 10.1016/j.mad.2022.111725 35995275

[B81] Liang X. M. Chen J. Zhang T. Liu J. Jiang X. J. Xu Z. Q. (2014). Cerebral hemorrhage increases plasma concentrations of noradrenalin and creatine kinase MB fraction with induction of cardiomyocyte damage in rats. Cell Biochem. Biophys. 70 (3), 1807–1811. 10.1007/s12013-014-0133-z 25022462

[B82] Lin H. B. Li F. X. Zhang J. Y. You Z. J. Xu S. Y. Liang W. B. (2021). Cerebral-cardiac syndrome and diabetes: Cardiac damage after ischemic stroke in diabetic state. Front. Immunol. 12, 737170. 10.3389/fimmu.2021.737170 34512671 PMC8430028

[B83] Linn J. Halpin A. Demaerel P. Ruhland J. Giese A. D. Dichgans M. (2010). Prevalence of superficial siderosis in patients with cerebral amyloid angiopathy. Neurology 74 (17), 1346–1350. 10.1212/WNL.0b013e3181dad605 20421578 PMC2875936

[B84] Liu C. Chen S. Zhang H. Chen Y. Gao Q. Chen Z. (2021). Bioinformatic analysis for potential biological processes and key targets of heart failure-related stroke. J. Zhejiang Univ. Sci. B 22 (9), 718–732. 10.1631/jzus.B2000544 34514752 PMC8435344

[B85] Ma S. Attarwala I. Y. Xie X. Q. (2019). SQSTM1/p62: A potential target for neurodegenerative disease. ACS Chem. Neurosci. 10 (5), 2094–2114. 10.1021/acschemneuro.8b00516 30657305 PMC6712989

[B86] Mandybur T. I. (1986). Cerebral amyloid angiopathy: The vascular pathology and complications. J. Neuropathol. Exp. Neurol. 45 (1), 79–90. 10.1097/00005072-198601000-00007 3941328

[B87] Mann D. L. (2015). Innate immunity and the failing heart: The cytokine hypothesis revisited. Circ. Res. 116 (7), 1254–1268. 10.1161/circresaha.116.302317 25814686 PMC4380242

[B88] Mann D. L. Kent R. L. Parsons B. Cooper G. (1992). Adrenergic effects on the biology of the adult mammalian cardiocyte. Circulation 85 (2), 790–804. 10.1161/01.cir.85.2.790 1370925

[B89] Marsh B. J. Williams-Karnesky R. L. Stenzel-Poore M. P. (2009). Toll-like receptor signaling in endogenous neuroprotection and stroke. Neuroscience 158 (3), 1007–1020. 10.1016/j.neuroscience.2008.07.067 18809468 PMC2674023

[B90] Mawuenyega K. G. Sigurdson W. Ovod V. Munsell L. Kasten T. Morris J. C. (2010). Decreased clearance of CNS beta-amyloid in Alzheimer's disease. Science 330 (6012), 1774doi. 10.1126/science.1197623 21148344 PMC3073454

[B91] Mehta P. Mehta J. (1979). Platelet function studies in coronary artery disease. V. Evidence for enhanced platelet microthrombus formation activity in acute myocardial infarction. Am. J. Cardiol. 43 (4), 757–760. 10.1016/0002-9149(79)90075-4 425912

[B92] Melone M. A. B. Dato C. Paladino S. Coppola C. Trebini C. Giordana M. T. (2018). Verapamil inhibits ser202/thr205 phosphorylation of tau by blocking TXNIP/ROS/p38 MAPK pathway. Pharm. Res. 35 (2), 44. 10.1007/s11095-017-2276-2 29404777

[B93] Merlini M. Wanner D. Nitsch R. M. (2016). Tau pathology-dependent remodelling of cerebral arteries precedes Alzheimer's disease-related microvascular cerebral amyloid angiopathy. Acta Neuropathol. 131 (5), 737–752. 10.1007/s00401-016-1560-2 26988843 PMC4835519

[B94] Mertes P. M. Carteaux J. P. Jaboin Y. Pinelli G. el Abassi K. Dopff C. (1994). Estimation of myocardial interstitial norepinephrine release after brain death using cardiac microdialysis. Transplantation 57 (3), 371–377. 10.1097/00007890-199402150-00010 8108872

[B95] Min J. Farooq M. U. Greenberg E. Aloka F. Bhatt A. Kassab M. (2009). Cardiac dysfunction after left permanent cerebral focal ischemia: The brain and heart connection. Stroke 40 (7), 2560–2563. 10.1161/strokeaha.108.536086 19443809 PMC2943768

[B96] Moreira P. I. Smith M. A. Zhu X. Nunomura A. Castellani R. J. Perry G. (2005). Oxidative stress and neurodegeneration. Ann. N. Y. Acad. Sci. 1043, 545–552. 10.1196/annals.1333.062 16037277

[B97] Mosconi M. G. Paciaroni M. Agnelli G. Marzano M. Alberti A. Venti M. (2021). SMASH-U classification: A tool for aetiology-oriented management of patients with acute haemorrhagic stroke. Intern Emerg. Med. 16 (1), 109–114. 10.1007/s11739-020-02330-2 32266689

[B98] Nagai M. Hoshide S. Kario K. (2010). The insular cortex and cardiovascular system: A new insight into the brain-heart axis. J. Am. Soc. Hypertens. 4 (4), 174–182. 10.1016/j.jash.2010.05.001 20655502

[B99] Nef H. M. Mollmann H. Hilpert P. Troidl C. Voss S. Rolf A. (2009). Activated cell survival cascade protects cardiomyocytes from cell death in Tako-Tsubo cardiomyopathy. Eur. J. Heart Fail 11 (8), 758–764. 10.1093/eurjhf/hfp076 19633102

[B100] Offner H. Subramanian S. Parker S. M. Afentoulis M. E. Vandenbark A. A. Hurn P. D. (2006). Experimental stroke induces massive, rapid activation of the peripheral immune system. J. Cereb. Blood Flow. Metab. 26 (5), 654–665. 10.1038/sj.jcbfm.9600217 16121126

[B101] Oppenheimer S. Cechetto D. (2016). The insular cortex and the regulation of cardiac function. Compr. Physiol. 6 (2), 1081–1133. 10.1002/cphy.c140076 27065176

[B102] Oppenheimer S. M. (1994). Neurogenic cardiac effects of cerebrovascular disease. Curr. Opin. Neurol. 7 (1), 20–24. 10.1097/00019052-199402000-00005 8173672

[B103] Oshima K. Uchikado H. Dickson D. W. (2008). Perivascular neuritic dystrophy associated with cerebral amyloid angiopathy in Alzheimer's disease. Int. J. Clin. Exp. Pathol. 1 (5), 403–408. 18787622 PMC2480573

[B104] Park G. Y. Jamerlan A. Shim K. H. (2019). Diagnostic and treatment approaches involving transthyretin in amyloidogenic diseases. Int. J. Mol. Sci. 20 (12), 2982. 10.3390/ijms20122982 31216785 PMC6628571

[B105] Pereira M. R. Leite P. E. (2016). The involvement of parasympathetic and sympathetic nerve in the inflammatory reflex. J. Cell Physiol. 231 (9), 1862–1869. 10.1002/jcp.25307 26754950

[B106] Piazza F. Greenberg S. M. Savoiardo M. Gardinetti M. Chiapparini L. Raicher I. (2013). Anti-amyloid β autoantibodies in cerebral amyloid angiopathy-related inflammation: Implications for amyloid-modifying therapies. Ann. Neurol. 73 (4), 449–458. 10.1002/ana.23857 23625526

[B107] Pirchl M. Humpel C. (2009).[Does acidosis in brain play a role in Alzheimer's disease? Neuropsychiatr 23 (3), 187–192. 19703385

[B108] Pullicino P. Homma S. (2010). Stroke in heart failure: Atrial fibrillation revisited? J. Stroke Cerebrovasc. Dis. 19 (1), 1–2. 10.1016/j.jstrokecerebrovasdis.2009.09.002 20123219

[B109] Revesz T. Holton J. L. Lashley T. Plant G. Rostagno A. Ghiso J. (2002). Sporadic and familial cerebral amyloid angiopathies. Brain Pathol. 12 (3), 343–357. 10.1111/j.1750-3639.2002.tb00449.x 12146803 PMC8095796

[B110] Richard M. A. Huan T. Ligthart S. Gondalia R. Jhun M. A. Brody J. A. (2017). DNA methylation analysis identifies loci for blood pressure regulation. Am. J. Hum. Genet. 101 (6), 888–902. 10.1016/j.ajhg.2017.09.028 29198723 PMC5812919

[B111] Roberson E. D. Halabisky B. Yoo J. W. Yao J. Chin J. Yan F. (2011). Amyloid-β/Fyn-induced synaptic, network, and cognitive impairments depend on tau levels in multiple mouse models of Alzheimer's disease. J. Neurosci. 31 (2), 700–711. 10.1523/jneurosci.4152-10.2011 21228179 PMC3325794

[B112] Roberson E. D. Scearce-Levie K. Palop J. J. Yan F. Cheng I. H. Wu T. (2007). Reducing endogenous tau ameliorates amyloid beta-induced deficits in an Alzheimer's disease mouse model. Science 316 (5825), 750–754. 10.1126/science.1141736 17478722

[B113] Roeben B. Maetzler W. Vanmechelen E. Schulte C. Heinzel S. Stellos K. (2016). Association of plasma Aβ40 peptides, but not Aβ42, with coronary artery disease and diabetes mellitus. J. Alzheimers Dis. 52 (1), 161–169. 10.3233/jad-150575 27003209

[B114] Ross J. A. Reyes B. A. S. Van Bockstaele E. J. (2019). Amyloid beta peptides, locus coeruleus-norepinephrine system and dense core vesicles. Brain Res. 1702, 46–53. 10.1016/j.brainres.2018.03.009 29577889 PMC6375485

[B115] Ruparelia N. Chai J. T. Fisher E. A. Choudhury R. P. (2017). Inflammatory processes in cardiovascular disease: A route to targeted therapies. Nat. Rev. Cardiol. 14 (3), 133–144. 10.1038/nrcardio.2016.185 27905474 PMC5525550

[B116] Rusanen M. Kivipelto M. Levalahti E. Laatikainen T. Tuomilehto J. Soininen H. (2014). Heart diseases and long-term risk of dementia and Alzheimer's disease: A population-based CAIDE study. J. Alzheimers Dis. 42 (1), 183–191. 10.3233/jad-132363 24825565

[B117] Sagris M. Theofilis P. Antonopoulos A. S. Oikonomou E. Paschaliori C. Galiatsatos N. (2021). Inflammation in coronary microvascular dysfunction. Int. J. Mol. Sci. 22 (24), 13471. 10.3390/ijms222413471 34948272 PMC8703507

[B118] Salvarani C. Hunder G. G. Morris J. M. Brown R. D. Jr Christianson T. Giannini C. (2013). Aβ-related angiitis: Comparison with CAA without inflammation and primary CNS vasculitis. Neurology 81 (18), 1596–1603. 10.1212/WNL.0b013e3182a9f545 24078731 PMC3806912

[B119] Samuels M. A. (1987). Neurogenic heart disease: A unifying hypothesis. Am. J. Cardiol. 60 (18), 15–19. 10.1016/0002-9149(87)90678-3 3321964

[B120] Sanna G. D. Nusdeo G. Piras M. R. Forteleoni A. Murru M. R. Saba P. S. (2019). Cardiac abnormalities in alzheimer disease: Clinical relevance beyond pathophysiological rationale and instrumental findings? JACC Heart Fail 7 (2), 121–128. 10.1016/j.jchf.2018.10.022 30704603

[B121] Schaich C. L. Maurer M. S. Nadkarni N. K. (2019). Amyloidosis of the brain and heart: Two sides of the same coin? JACC Heart Fail 7 (2), 129–131. 10.1016/j.jchf.2018.12.014 30704604 PMC6515925

[B122] Schönhofen P. de Medeiros L. M. Chatain C. P. Bristot I. J. Klamt F. (2014). Cofilin/actin rod formation by dysregulation of cofilin-1 activity as a central initial step in neurodegeneration. Mini Rev. Med. Chem. 14 (5), 393–400. 10.2174/1389557514666140506161458 24813767

[B123] Semenas E. Sharma H. S. Wiklund L. (2014). Adrenaline increases blood-brain-barrier permeability after haemorrhagic cardiac arrest in immature pigs. Acta Anaesthesiol. Scand. 58 (5), 620–629. 10.1111/aas.12293 24580085

[B124] Shi K. Tian D. C. Li Z. G. Ducruet A. F. Lawton M. T. Shi F. D. (2019). Global brain inflammation in stroke. Lancet Neurol. 18 (11), 1058–1066. 10.1016/s1474-4422(19)30078-x 31296369

[B125] Shichita T. Hasegawa E. Kimura A. Morita R. Sakaguchi R. Takada I. (2012). Peroxiredoxin family proteins are key initiators of post-ischemic inflammation in the brain. Nat. Med. 18 (6), 911–917. 10.1038/nm.2749 22610280

[B126] Shigematsu K. McGeer P. L. Walker D. G. Ishii T. McGeer E. G. (1992). Reactive microglia/macrophages phagocytose amyloid precursor protein produced by neurons following neural damage. J. Neurosci. Res. 31 (3), 443–453. 10.1002/jnr.490310306 1640496

[B127] Shishehbor M. H. Alves C. Rajagopal V. (2007). Inflammation: Implications for understanding the heart-brain connection. Cleve Clin. J. Med. 74 (1), S37–S41. 10.3949/ccjm.74.suppl_1.s37 17455542

[B128] Shityakov S. Hayashi K. Stork S. Scheper V. Lenarz T. Forster C. Y. (2021). The conspicuous link between ear, brain and heart-could neurotrophin-treatment of age-related hearing loss help prevent Alzheimer's disease and associated amyloid cardiomyopathy? Biomolecules 11 (6), 900. 10.3390/biom11060900 34204299 PMC8235707

[B129] Silva C. S. Eira J. Ribeiro C. A. Oliveira A. Sousa M. M. Cardoso I. (2017). Transthyretin neuroprotection in Alzheimer's disease is dependent on proteolysis. Neurobiol. Aging 59, 10–14. 10.1016/j.neurobiolaging.2017.07.002 28780366

[B130] Sposato L. A. Hilz M. J. Aspberg S. Murthy S. B. Bahit M. C. Hsieh C. Y. (2020). Post-stroke cardiovascular complications and neurogenic cardiac injury: JACC state-of-the-art review. J. Am. Coll. Cardiol. 76 (23), 2768–2785. 10.1016/j.jacc.2020.10.009 33272372

[B131] Stakos D. A. Stamatelopoulos K. Bampatsias D. Sachse M. Zormpas E. Vlachogiannis N. I. (2020). The Alzheimer's disease amyloid-beta hypothesis in cardiovascular aging and disease: JACC focus seminar. J. Am. Coll. Cardiol. 75 (8), 952–967. 10.1016/j.jacc.2019.12.033 32130931 PMC7042886

[B132] Stamatelopoulos K. Pol C. J. Ayers C. Georgiopoulos G. Gatsiou A. Brilakis E. S. (2018). Amyloid-Beta (1-40) peptide and subclinical cardiovascular disease. J. Am. Coll. Cardiol. 72 (9), 1060–1061. 10.1016/j.jacc.2018.06.027 30139434 PMC6467498

[B133] Subramanian K. Gianni D. Balla C. Assenza G. E. Joshi M. Semigran M. J. (2015). Cofilin-2 phosphorylation and sequestration in myocardial aggregates: Novel pathogenetic mechanisms for idiopathic dilated cardiomyopathy. J. Am. Coll. Cardiol. 65 (12), 1199–1214. 10.1016/j.jacc.2015.01.031 25814227 PMC4379451

[B134] Sun X. Y. Li L. J. Dong Q. X. Zhu J. Huang Y. R. Hou S. J. (2021). Rutin prevents tau pathology and neuroinflammation in a mouse model of Alzheimer's disease. J. Neuroinflammation 18 (1), 131. 10.1186/s12974-021-02182-3 34116706 PMC8196535

[B135] Szabadi E. (2013). Functional neuroanatomy of the central noradrenergic system. J. Psychopharmacol. 27 (8), 659–693. 10.1177/0269881113490326 23761387

[B136] Tajima H. Kawasumi M. Chiba T. Yamada M. Yamashita K. Nawa M. (2005). A humanin derivative, S14G-HN, prevents amyloid-beta-induced memory impairment in mice. J. Neurosci. Res. 79 (5), 714–723. 10.1002/jnr.20391 15678515

[B137] Tanskanen M. Peuralinna T. Polvikoski T. Notkola I. L. Sulkava R. Hardy J. (2008). Senile systemic amyloidosis affects 25% of the very aged and associates with genetic variation in alpha2-macroglobulin and tau: A population-based autopsy study. Ann. Med. 40 (3), 232–239. 10.1080/07853890701842988 18382889

[B138] Terwel D. Muyllaert D. Dewachter I. Borghgraef P. Croes S. Devijver H. (2008). Amyloid activates GSK-3beta to aggravate neuronal tauopathy in bigenic mice. Am. J. Pathol. 172 (3), 786–798. 10.2353/ajpath.2008.070904 18258852 PMC2258274

[B139] Tiwari S. Atluri V. Kaushik A. Yndart A. Nair M. (2019). Alzheimer's disease: Pathogenesis, diagnostics, and therapeutics. Int. J. Nanomedicine 14, 5541–5554. 10.2147/ijn.S200490 31410002 PMC6650620

[B140] Troncone L. Luciani M. Coggins M. Wilker E. H. Ho C. Y. Codispoti K. E. (2016). Aβ amyloid pathology affects the hearts of patients with Alzheimer's disease: Mind the heart. J. Am. Coll. Cardiol. 68 (22), 2395–2407. 10.1016/j.jacc.2016.08.073 27908343 PMC5142757

[B141] Tsubaki H. Tooyama I. Walker D. G. (2020). Thioredoxin-interacting protein (TXNIP) with focus on brain and neurodegenerative diseases. Int. J. Mol. Sci. 21 (24), 9357. 10.3390/ijms21249357 33302545 PMC7764580

[B142] Tublin J. M. Adelstein J. M. Del Monte F. Combs C. K. Wold L. E. (2019). Getting to the heart of alzheimer disease. Circ. Res. 124 (1), 142–149. 10.1161/circresaha.118.313563 30605407 PMC6319653

[B143] Vidal R. Calero M. Piccardo P. Farlow M. R. Unverzagt F. W. Mendez E. (2000a). Senile dementia associated with amyloid beta protein angiopathy and tau perivascular pathology but not neuritic plaques in patients homozygous for the APOE-epsilon4 allele. Acta Neuropathol. 100 (1), 1–12. 10.1007/s004010051186 10912914

[B144] Vidal R. Revesz T. Rostagno A. Kim E. Holton J. L. Bek T. (2000b). A decamer duplication in the 3' region of the BRI gene originates an amyloid peptide that is associated with dementia in a Danish kindred. Proc. Natl. Acad. Sci. U. S. A. 97 (9), 4920–4925. 10.1073/pnas.080076097 10781099 PMC18333

[B145] Viswanathan A. Greenberg S. M. (2011). Cerebral amyloid angiopathy in the elderly. Ann. Neurol. 70 (6), 871–880. 10.1002/ana.22516 22190361 PMC4004372

[B146] Wang B. Cai Z. Liu B. Liu Z. Zhou X. Dong N. (2017). RAGE deficiency alleviates aortic valve calcification in ApoE(-/-) mice via the inhibition of endoplasmic reticulum stress. Biochim. Biophys. Acta Mol. Basis Dis. 1863 (3), 781–792. 10.1016/j.bbadis.2016.12.012 28024939

[B147] Wang B. Lin H. Q. Li F. Mao Z. F. Dong N. G. (2020). Aβ40 promotes the osteoblastic differentiation of aortic valve interstitial cells through the RAGE pathway. Curr. Med. Sci. 40 (5), 931–936. 10.1007/s11596-020-2264-3 33123906

[B148] Wang J. Doré S. (2007). Inflammation after intracerebral hemorrhage. J. Cereb. Blood Flow. Metab. 27 (5), 894–908. 10.1038/sj.jcbfm.9600403 17033693

[B149] Weber S. A. Patel R. K. Lutsep H. L. (2018). Cerebral amyloid angiopathy: Diagnosis and potential therapies. Expert Rev. Neurother. 18 (6), 503–513. 10.1080/14737175.2018.1480938 29792540

[B150] Weller R. O. Subash M. Preston S. D. Mazanti I. Carare R. O. (2008). Perivascular drainage of amyloid-beta peptides from the brain and its failure in cerebral amyloid angiopathy and Alzheimer's disease. Brain Pathol. 18 (2), 253–266. 10.1111/j.1750-3639.2008.00133.x 18363936 PMC8095597

[B151] Whitnall M. H. (1993). Regulation of the hypothalamic corticotropin-releasing hormone neurosecretory system. Prog. Neurobiol. 40 (5), 573–629. 10.1016/0301-0082(93)90035-q 8484004

[B152] Willem M. Tahirovic S. Busche M. A. Ovsepian S. V. Chafai M. Kootar S. (2015). η-Secretase processing of APP inhibits neuronal activity in the hippocampus. Nature 526 (7573), 443–447. 10.1038/nature14864 26322584 PMC6570618

[B153] Willis M. S. Patterson C. (2013). Proteotoxicity and cardiac dysfunction-Alzheimer's disease of the heart? N. Engl. J. Med. 368 (5), 455–464. 10.1056/NEJMra1106180 23363499

[B154] Yamashita T. Ando Y. Ueda M. Nakamura M. Okamoto S. Zeledon M. E. (2008). Effect of liver transplantation on transthyretin Tyr114Cys-related cerebral amyloid angiopathy. Neurology 70 (2), 123–128. 10.1212/01.wnl.0000287089.28847.b5 18180441

[B155] You Y. Perkins A. Cisternas P. Munoz B. Taylor X. (2019). Tau as a mediator of neurotoxicity associated to cerebral amyloid angiopathy. Acta Neuropathol. Commun. 7 (1), 26. 10.1186/s40478-019-0680-z 30808415 PMC6390363

[B156] Zhang Y. Huang J. Yang X. Sun X. Xu Q. Wang B. (2017). Altered Expression of TXNIP in the peripheral leukocytes of patients with coronary atherosclerotic heart disease. Med. Baltim. 96 (49), e9108. 10.1097/md.0000000000009108 PMC5728958 29245343

[B157] Zhang Z. Song M. Liu X. Su Kang S. Duong D. M. Seyfried N. T. (2015). Delta-secretase cleaves amyloid precursor protein and regulates the pathogenesis in Alzheimer's disease. Nat. Commun. 6, 8762. 10.1038/ncomms9762 26549211 PMC4659940

[B158] Zhou H. Gao F. Yang X. Lin T. Li Z. Wang Q. (2022). Endothelial BACE1 impairs cerebral small vessels via tight junctions and eNOS. Circ. Res. 130 (9), 1321–1341. 10.1161/circresaha.121.320183 35382554

[B159] Zhou Y. Wang Y. Wang J. Anne Stetler R. Yang Q. W. (2014). Inflammation in intracerebral hemorrhage: From mechanisms to clinical translation. Prog. Neurobiol. 115, 25–44. 10.1016/j.pneurobio.2013.11.003 24291544

